# Genomic analysis reveals an exogenous viral symbiont with dual functionality in parasitoid wasps and their hosts

**DOI:** 10.1371/journal.ppat.1009069

**Published:** 2020-11-30

**Authors:** Kelsey A. Coffman, Gaelen R. Burke

**Affiliations:** Department of Entomology, University of Georgia, Athens, Georgia, United States of America; Pennsylvania State University, UNITED STATES

## Abstract

Insects are known to host a wide variety of beneficial microbes that are fundamental to many aspects of their biology and have substantially shaped their evolution. Notably, parasitoid wasps have repeatedly evolved beneficial associations with viruses that enable developing wasps to survive as parasites that feed from other insects. Ongoing genomic sequencing efforts have revealed that most of these virus-derived entities are fully integrated into the genomes of parasitoid wasp lineages, representing endogenous viral elements (EVEs) that retain the ability to produce virus or virus-like particles within wasp reproductive tissues. All documented parasitoid EVEs have undergone similar genomic rearrangements compared to their viral ancestors characterized by viral genes scattered across wasp genomes and specific viral gene losses. The recurrent presence of viral endogenization and genomic reorganization in beneficial virus systems identified to date suggest that these features are crucial to forming heritable alliances between parasitoid wasps and viruses. Here, our genomic characterization of a mutualistic poxvirus associated with the wasp *Diachasmimorpha longicaudata*, known as Diachasmimorpha longicaudata entomopoxvirus (DlEPV), has uncovered the first instance of beneficial virus evolution that does not conform to the genomic architecture shared by parasitoid EVEs with which it displays evolutionary convergence. Rather, DlEPV retains the exogenous viral genome of its poxvirus ancestor and the majority of conserved poxvirus core genes. Additional comparative analyses indicate that DlEPV is related to a fly pathogen and contains a novel gene expansion that may be adaptive to its symbiotic role. Finally, differential expression analysis during virus replication in wasps and fly hosts demonstrates a unique mechanism of functional partitioning that allows DlEPV to persist within and provide benefit to its parasitoid wasp host.

## Author summary

Viruses have repeatedly formed long-term associations with insects called parasitoid wasps, which grow as parasites within other insect hosts. While these viruses were once pathogenic, they have since been co-opted by parasitoid wasps to benefit the survival of wasp offspring during parasitism. The genomes of most identified beneficial viruses are fully integrated into the genomes of the parasitoid wasps that produce them. Because these virus-derived entities have lost the ability to exist apart from their associated wasps, they are considered endogenous viral elements (EVEs) of the wasps rather than mutualistic symbionts. We sequenced the genome of the beneficial parasitoid virus Diachasmimorpha longicaudata entomopoxvirus (DlEPV) and found that its genome is not integrated into the genome of *Diachasmimorpha longicaudata* wasps and has largely retained the genomic structure of its pathogenic ancestor. Given the importance of viral genome integration in the overall stability of parasitoid wasp-EVE systems, we identified a novel strategy used by DlEPV to maintain its relationship with *D*. *longicaudata* despite its lack of endogenization. Our findings in this study demonstrate the first instance of a mutualistic viral symbiont in insects and provide new insight into the means through which beneficial viruses can arise.

## Introduction

Microbial symbionts have been increasingly identified as major drivers of animal evolution due to the novel capabilities microbes provide to their hosts and the speed at which symbiosis can cause adaptive change in animal lineages [1,2]. Insects, in particular, have repeatedly formed symbiotic alliances with microbes that highly vary with respect to taxonomic classification, localization on or within the insect, transmission strategies, and phenotypic traits provided to the insect [3]. Bacterial symbionts have been the primary focus of study for many insect groups, such as plant sap-feeders, blood-feeders, and social insects [4–6]. However, parasitoid wasps, whose young are obligate parasites of other arthropods, are better known for their numerous associations with viruses [7–9]. Parasitoid wasp lineages have repeatedly acquired heritable viruses in conjunction with evolutionary arms race dynamics between wasps and their hosts [10–12]. Many of these associations are extraordinary examples of endogenous viral elements (EVEs) within wasp genomes, in which components of viral machinery are retained from their pathogenic ancestors to produce virus or virus-like particles within wasp ovaries [13,14]. The resulting virus-derived particles accompany wasp eggs when delivered into host insects and can function to protect parasitoid eggs from attack by the host immune system and/or actively disrupt host developmental and immunological pathways [10,15,16].

Rather than existing in a wasp genome as a contiguous region of proviral DNA, parasitoid EVEs share an unconventional genomic architecture characterized by the dispersal of viral genes to separate regions of the wasp genome [17–22]. Key virus replication genes have also been lost in all cases for which the viral ancestor is known, which implies that wasp genes are instead needed to complete virus particle production [23]. These genomic anomalies have three major consequences that are thought to be important adaptations in parasitoid-EVE associations. First, permanent integration of the viral genome into the wasp genome ensures viral transmission to future wasp generations. Second, viral gene dispersal and gene loss forfeits EVE autonomy over their own propagation, allowing for strict regulation of virus replication by the wasp. Third, virus particles produced in wasp tissue do not contain the necessary genes for further replication outside of the wasp. The functional outcome of these genomic features is most clearly understood in the polydnaviruses (PDVs), a group of ancient EVEs formed from multiple, independent viral acquisition events [17,18]. Due to their unusual genome organization, PDVs contain a dual functionality that is effectively split between two insects: PDV replication occurs exclusively in wasp tissue, while PDV virulence is confined to parasitized host tissue [24,25]. This separation of virus function promotes stability within these associations, because it minimizes wasp-virus conflict and establishes an interdependency for survival, in which wasp offspring depend on PDV virulence within the host, and PDVs depend on wasps for transmission and amplification [26,27]. Furthermore, the recurrent observation of this distinctive genomic architecture in more recently acquired EVEs supports the notion that viral genome integration and reorganization is fundamental to the persistence of wasp-virus mergers [21,22].

However, additional examples of heritable viruses have been identified in parasitoid lineages that are of unique viral origin and may deviate from this pattern. An ascovirus carried by the wasp *Diadromus pulchellus*, named Diadromus pulchellus toursvirus (DpTV), contains a circular, episomal DNA genome present in the nuclei of wasp cells, and when DpTV virions infect the caterpillar hosts of *D*. *pulchellus*, the virus inhibits the host melanization response during an immune challenge [28–30]. Additionally, a heritable iflavirus discovered within *Dinocampus coccinellae* parasitoid wasps, known as Dinocampus coccinellae paralysis virus (DcPV), contains an exogenous RNA viral genome that replicates within the neural tissue of coccinellid beetle hosts of the wasps during parasitism. This viral activity is thought to cause a behavioral manipulation within the host, in which parasitized beetles will guard the parasitoid pupa against predation [31]. While these examples provide suggestive evidence that the virus in each case is beneficial for the parasitoid that transmits it, neither example has been experimentally shown to provide a direct fitness benefit for its associated wasp. An effective method to determine whether heritable viruses are truly mutualistic for wasps is to remove the virus population from wasps and compare the success of “cured” wasps to those with a normal viral load. Lower survivorship of wasp progeny when not accompanied by virus is strong evidence that the virus provides a net benefit to wasp fitness.

This has been recently demonstrated for *Diachasmimorpha longicaudata* parasitoid wasps and the heritable poxvirus female wasps maintain within their venom gland, known as Diachasmimorpha longicaudata entomopoxvirus (DlEPV) [32,33]. We showed that DlEPV is vertically transmitted to each wasp generation within oviposited wasp eggs and provides a considerable boost to wasp survival during parasitism within *Anastrepha suspensa* fruit fly hosts, because wasps reared without DlEPV survive at a drastically reduced rate compared to wasps with a typical viral load [34]. DlEPV is currently the only mutualistic poxvirus to be identified in parasitoid wasps, and unlike EVEs, DlEPV can replicate within both wasps and fruit fly hosts of the wasps [34]. Therefore, DlEPV appears to have replicative autonomy within both insects, suggesting that this virus retains more features from its pathogenic ancestor than other parasitoid viral elements. Despite these ancestral characteristics, we have also demonstrated that DlEPV replication is highly virulent within host fly tissue, while replication within wasp tissue has no observable pathogenic effects [34]. These results imply that DlEPV utilizes a similar strategy of functional partitioning to that observed in parasitoid EVEs. DlEPV is therefore unique in that it maintains features of both an autonomous viral pathogen and a beneficial viral symbiont. In this study, we sequenced the complete DlEPV genome to ascertain whether DlEPV shares the genomic architecture of parasitoid EVEs and to determine how DlEPV has evolved in comparison to other poxviruses, including the identification of its closest known relative. Using the results from our comparative analyses, we then performed a functional genomic investigation to elucidate the novel means through which DlEPV may achieve its beneficial relationship with *D*. *longicaudata*.

## Results

### Sequencing and assembly of the DlEPV genome

#### The DlEPV genome is non-endogenous

**Fig 1:**
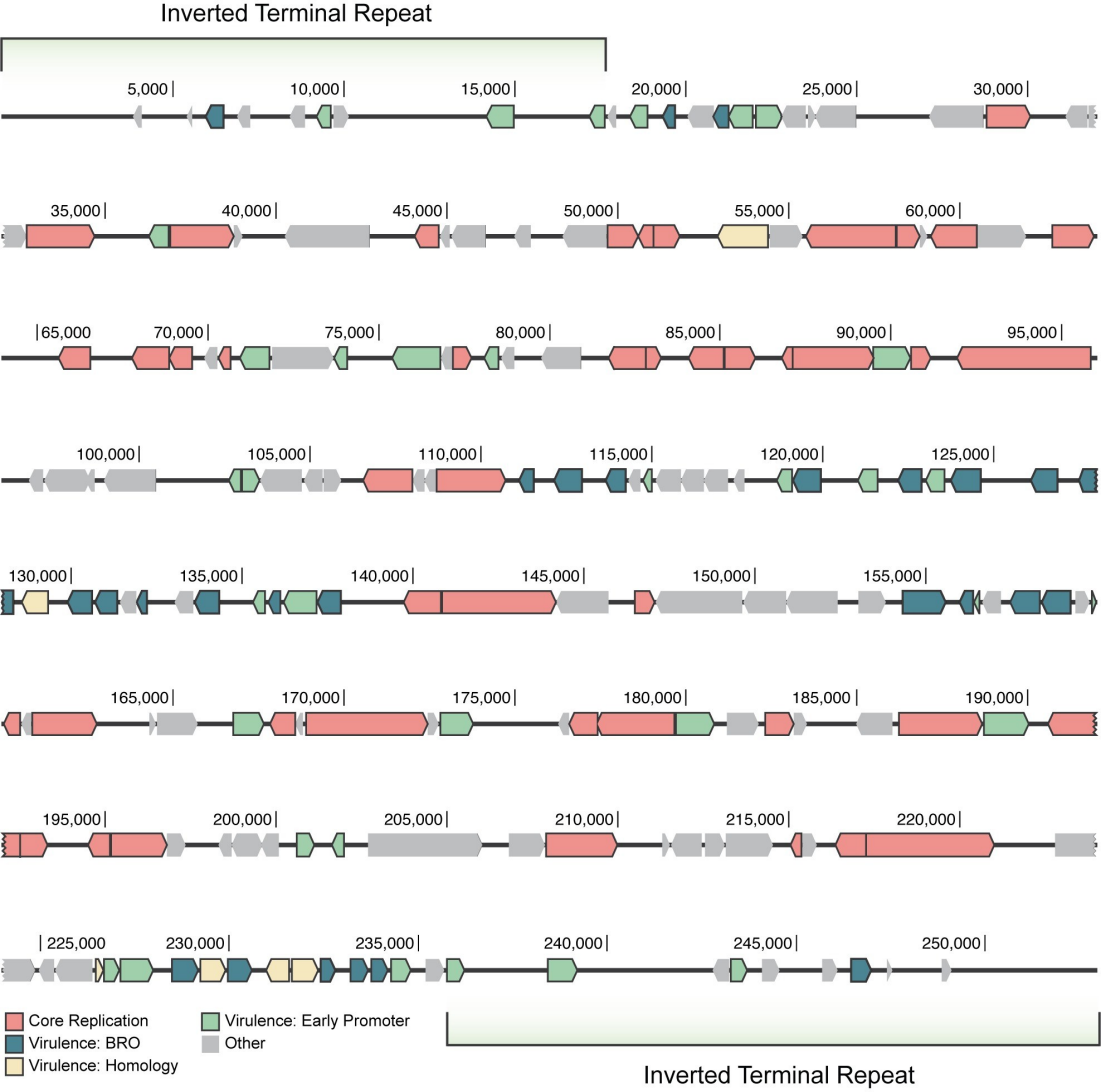
Linear map of the DlEPV genome. Each arrow indicates the genomic position of a DlEPV ORF, and the direction of the arrow corresponds to its strand orientation. Arrows are colored based on the putative functional category of each ORF as defined in the legend at the bottom of the map. Core Replication refers to the 45 poxvirus core genes identified in the DlEPV genome. Virulence: BRO refers to the 27 DlEPV BRO genes, Virulence: Homology indicates the 6 ORFs with similarity to known virulence genes, and Virulence: Early Promoter are the additional 34 putative virulence genes based on the presence of the conserved EPV early promoter sequence and no other assigned function.

Poxviruses are large DNA viruses that infect vertebrates (chordopoxviruses, or CPVs), as well as insects (entomopoxviruses, or EPVs) [35]. The study of poxviruses has historically focused on CPVs and the prototype CPV, known as vaccinia virus (VACV), due to the societal impact of smallpox [36]. EPVs have been comparatively neglected but function similarly to CPVs in many ways, while exhibiting differences that can often be attributed to the biology of their insect hosts [37]. Both CPV and EPV genomes exist as linear, double-stranded DNA (dsDNA) molecules that contain a hairpin loop at each terminus. The two extreme ends of the genome consist of sequence repeats that are inversions of one another, known as inverted terminal repeats (ITRs), while the genome interior contains the majority of viral genes [38]. The DlEPV genome sequence, obtained from high-throughput sequencing of *D*. *longicaudata* venom gland DNA, was assembled into a single 253 kilobase (kb) contiguous sequence, including two 17 kb ITR regions and 193 open reading frames (ORFs) (Fig 1 and S1 Table). The contiguity and lack of flanking wasp genes in our assembly implies that the DlEPV genome is not endogenous within the wasp genome. Normalized quantitative PCR (qPCR) data of viral abundance in wasp tissue also support this finding by showing that the number of DlEPV genome copies is less than the number of wasp genome copies for several tissues, developmental stages, and all male wasps (S1 Fig).

#### The DlEPV genome has abnormally low coding density and high GC content

The majority of publicly available EPV genomes are from lepidopteran (moth and butterfly) poxviruses: Amsacta moorei entomopoxvirus (AMEV), Adoxophyes honmai entomopoxvirus (AHEV), Choristoneura biennis entomopoxvirus (CBEV), Choristoneura rosaceana entomopoxvirus (CREV), and Mythimna separata entomopoxvirus (MySEV) [39,40]. Orthopteran (grasshopper) and coleopteran (beetle) poxvirus genomes contain single representatives: Melanoplus sanguinipes entomopoxvirus (MSEV) and Anomala cuprea entomopoxvirus (ACEV), respectively [41,42]. Recently, two additional EPV sequences have been reported. A partial poxvirus genome sequence identified within the argentine ant, named Linepithema humile entomopoxvirus 1 (LHEV), represents the first hymenopteran (ant, wasp, and bee) poxvirus to be sequenced [43]. In addition, a complete poxvirus genome obtained from *Drosophila melanogaster*, known as Yalta virus, represents the first sequenced dipteran (fly) poxvirus [44].

DlEPV, in comparison to these other EPVs, has a similar overall genome length, ITR length, and ORF number (S2 Table). However, the DlEPV genome is peculiar with respect to its coding capacity and GC content. DlEPV is extremely gene-sparse relative to its genome size and contains a heavily reduced coding density of 65.1% compared to the 89.9% ± 3.0% SD coding density of other EPV genomes (S2 Table). This reduced gene density is an exception to poxvirus genomes, in general, which are highly compact with a dense array of non-overlapping genes [38]. The nucleotide composition of the DlEPV genome also varies compared to other EPV genomes, which consistently exhibit the most severe AT-bias found in the poxvirus family [45]. The GC content of the DlEPV genome at 30.1% is substantially higher than the average 20.5% ± 2.4% SD of its EPV relatives (S2 Table). Since viral GC content can be correlated to the GC of the host genome [46], we also estimated *D*. *longicaudata* and *A*. *suspensa* genome nucleotide composition using transcriptomes produced for a subsequent differential expression analysis (see Functional Genomic Analysis of DlEPV). Assembled *D*. *longicaudata* transcripts had 40.7% GC overall, and *A*. *suspensa* fly hosts contained transcripts with a GC content of 39.7%.

### DlEPV genome annotation

#### DlEPV contains most poxvirus core genes

We next annotated the DlEPV genome to assess its completeness compared to other poxviruses. The central region of the linear poxvirus genome generally contains genes that are required for virus replication, including the 49 core genes conserved among all sequenced poxviruses [45,47,48]. We were able to identify the majority of poxvirus core genes in the DlEPV genome, with the exception of the following four genes: the heparin binding surface protein (VACV core gene H3L), a virion core protein (E6R), the NlpC/P60 superfamily protein (G6R), and a RNA polymerase subunit (A29L) [49] (S1 Table). VACV core genes H3L and E6R are both required for the correct assemblage of mature virions, a process known as morphogenesis [50–54]. G6R is unique among the poxvirus core gene set, as its protein product is not required for VACV replication *in vitro* but instead is involved in virulence [55]. A29L encodes the 35 kDa RNA polymerase subunit (RPO35), one of five conserved subunits of the poxvirus RNA polymerase holoenzyme responsible for viral gene transcription [56]. We utilized our previously reported transcriptome of the *D*. *longicaudata* venom gland [34] to determine whether these four genes had been transferred from the DlEPV genome to the *D*. *longicaudata* genome. Endogenized PDV replication genes were first identified in PDV-producing wasps using transcriptome sequencing of wasp ovary tissue collected during PDV replication [17,18]. We therefore hypothesized that DlEPV transcripts with sequence similarity to the undetected genes would be present during virus replication in the venom gland if these genes were endogenous. However, our transcriptome searches yielded no hits to the aforementioned genes.

#### DlEPV is most closely related to a *Drosophila* poxvirus

Due to the exogenous state of the DlEPV genome and its relatively complete set of core genes, DlEPV appears to be more biologically similar to its viral progenitor than has been observed of parasitoid EVEs. This level of genomic preservation led us to investigate the origin of DlEPV among other poxviruses through phylogenetic reconstruction and identification of its closest relative. Because DlEPV replicates in both a parasitoid wasp and the wasp’s fruit fly hosts, it is likely that DlEPV originated as either a parasitoid pathogen or as a fly pathogen. The two most recently published EPV genomes, LHEV and Yalta virus, could therefore give more context on the origin of DlEPV.

**Fig 2:**
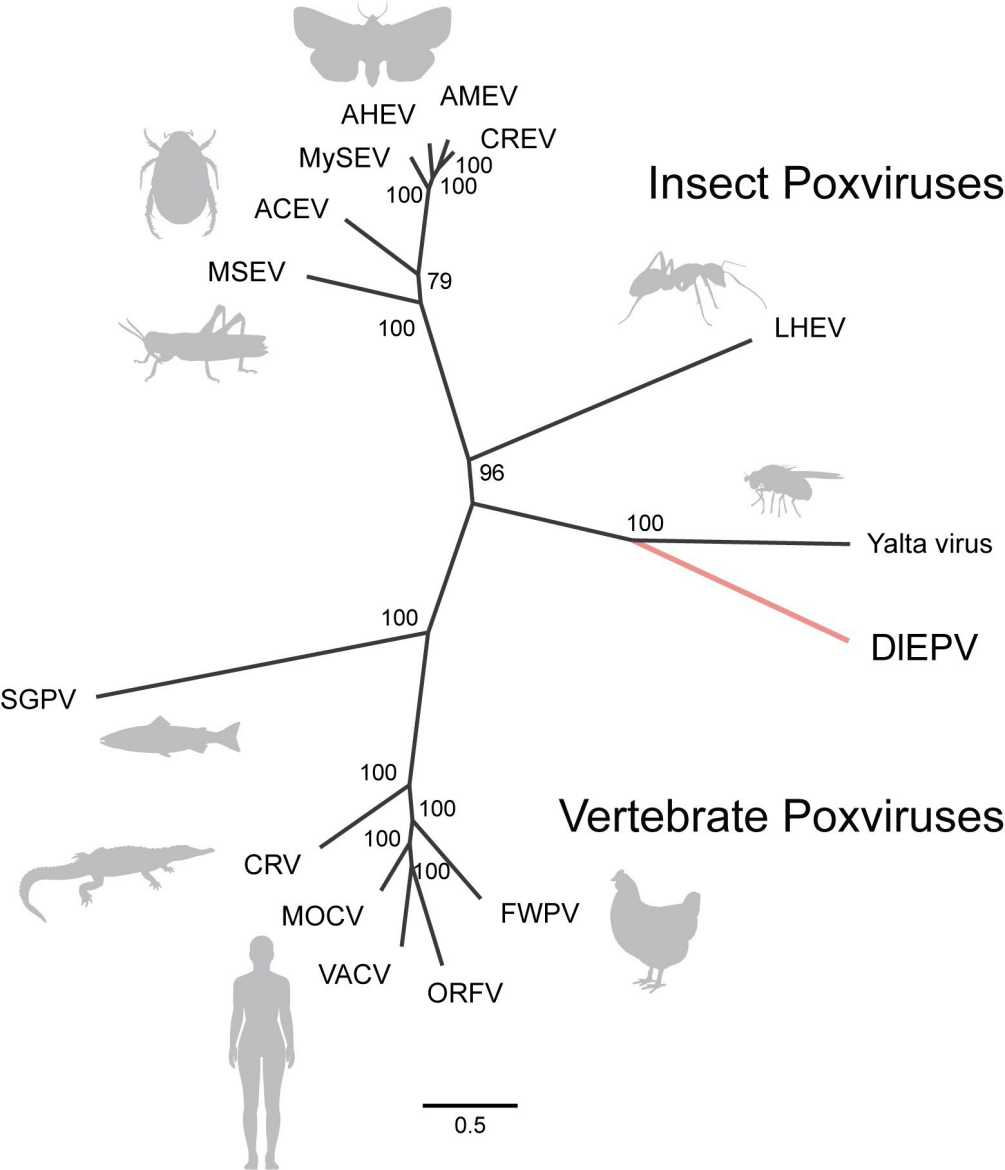
Poxvirus core gene phylogeny demonstrates that the fly-infecting Yalta virus is the closest known relative of DlEPV. Phylogenetic tree constructed from a maximum likelihood analysis using the concatenated amino acid multiple sequence alignment from 16 conserved poxvirus core genes. Node support (%) was inferred with 1,000 bootstrap iterations. Insect and vertebrate poxvirus orthologs used to build the phylogeny are indicated in S3 Table. Genome abbreviations are as defined in the Results section, and accessions are included in the Materials and Methods section.

We generated a maximum likelihood (ML) phylogeny using 16 concatenated poxvirus core genes from all sequenced EPVs and the following CPVs to test the two hypotheses: VACV, orf virus (ORFV), molluscum contagiosum virus (MOCV), fowlpox virus (FWPV), crocodilepox virus (CRV), and salmon gill poxvirus (SGPV) (Fig 2 and S3 Table). The placement of the 7 originally sequenced EPVs (MSEV, AMEV, AHEV, MySEV, CREV, CBEV, and ACEV) on the tree shows concordance with the higher phylogenetic relationships of their insect hosts, which is consistent with previous EPV phylogenetic analyses [37,40]. In contrast, LHEV and Yalta virus show a clear divergence from other EPVs [44]. The inclusion of DlEPV in our phylogeny revealed that it shares a more recent common ancestor with Yalta virus than LHEV, suggesting that DlEPV is more likely derived from a fly pathogen rather than a parasitoid pathogen (Fig 2). The shared common ancestry between DlEPV and Yalta virus was maintained in an expanded 44 core gene phylogeny that excluded the partial LHEV genome, and the overall tree topology was robust to Bayesian inference analysis (S2 Fig). While the position of the DlEPV/Yalta virus clade appears to bridge the gap between EPVs and CPVs in the unrooted phylogeny, the inclusion of members of the sister group to poxviruses, known as Asfarviridae [57], as an outgroup in a 10 poxvirus core gene phylogeny confirmed that DlEPV and Yalta virus are, in fact, more closely related to EPVs than to CPVs (S2 Fig).

We further investigated similarities between the DlEPV and Yalta virus genomes by searching for additional orthologous genes shared among them. We determined that 44 of the 49 poxvirus core genes are shared between the DlEPV and Yalta virus genomes (S3 Table). Interestingly, 2 of the 3 “missing” genes in the Yalta virus genome were also not detected in the DlEPV genome [44]. This suggests that the absence, or more likely, extreme sequence divergence of these genes is lineage-specific to fly poxviruses, rather than due to genome incompleteness. In addition to the 44 core genes shared between the two genomes, we found 24 single-copy orthologs and 3 orthologs that had undergone duplication in either genome (S1 Table). Most of these orthologous groups are of unknown function, while some have putative functions that are not found in other EPVs and therefore, may be unique to fly poxviruses. These include a ribonucleotide reductase large subunit (DLEV028/Yalta121), an alpha/beta fold hydrolase (DLEV038/Yalta165), and a type II topoisomerase (DLEV158/Yalta014).

**Fig 3:**
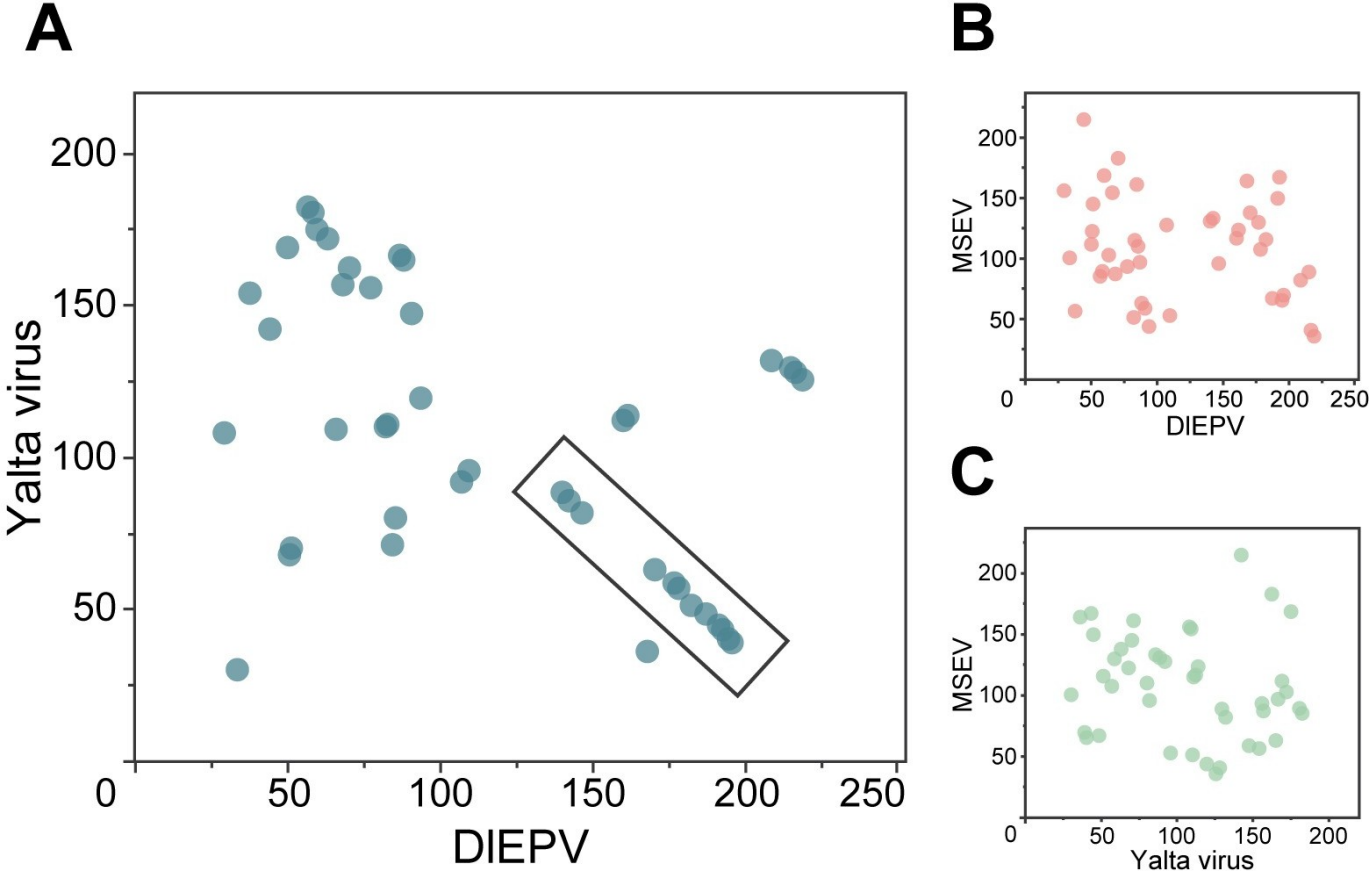
Core gene synteny further supports the close relationship between DlEPV and Yalta virus. Dot plots show the relative genomic location of the 44 poxvirus core genes shared between the DlEPV and Yalta virus genomes when compared to (A) one another, or (B-C) when either is compared to MSEV. Each dot represents a homologous core gene, and axes indicate the genomic position in kilobases. Box in panel A highlights a highly syntenic region between DlEPV and Yalta virus core genes.

While many CPV genomes have a highly conserved gene order [58], this colinear pattern does not hold true for EPVs, which display little synteny with CPVs or EPVs from separate host genera [39,40,42]. We investigated genome synteny between DlEPV and Yalta virus by generating two-dimensional dot plots comparing the genomic positions of their shared 44 core genes (Fig 3). The DlEPV-Yalta virus dot plot revealed a moderate amount of synteny between the two viral genomes. In particular, a large syntenic region of approximately 50 kb was identified, as indicated by the negative linear arrangement of orthologs in the lower-right quadrant of the plot (Fig 3A). This partial synteny further supports a closer relationship between DlEPV and Yalta virus, because both genomes have relatively low synteny when compared to the next closest relative MSEV (Fig 3B and 3C).

#### The DlEPV genome contains a novel BRO gene expansion

In contrast to the more conserved center of the typical poxvirus genome, the exterior regions contain a variable assortment of virulence genes, which are involved in host interactions [45,59]. Multigene families are common components of large DNA viral genomes that likely represent lineage-specific adaptations resulting from coevolution between the virus and its host [60]. One such family, known as baculovirus repeated ORF (BRO) proteins, are found in many insect DNA viruses, including baculoviruses, iridoviruses, ascoviruses, and EPVs [61]. The function of BRO proteins remains unclear, although they localize to the nuclei of insect cells infected with the baculovirus Bombyx mori nucleopolyhedrovirus [62], and the characteristic N-terminal BRO domain can bind DNA [63]. Therefore, it has been proposed that BRO proteins may play a role in virulence through the regulation of host DNA transcription and/or replication [63]. BRO genes are consistently found in the terminal regions of EPV genomes amongst other virulence genes [39–42]. Strikingly, the DlEPV genome contains 27 BRO genes, which is >3 times the average quantity found in other EPVs (7.6 gene copies ± 4.8 SD) (S4 Table). Furthermore, 14 of the DlEPV BRO genes are clustered together in the most central 30 kb of the genome, while 4 additional BRO genes form a secondary cluster within 17 kb of the primary cluster (Fig 1). The uniform strand orientation and relative size of BRO genes in the primary cluster suggests that this region represents a large gene family expansion via tandem duplication events.

#### Few other homologous virulence genes were identified

Three DlEPV virulence genes could be identified by sequence similarity to virulence genes in other viruses, including a thymidylate kinase (DLEV176), a thymidine kinase (DLEV178), and a F-box protein (DLEV179) (S1 Table). Thymidine and thymidylate kinases are ubiquitous genes involved in DNA biosynthesis of both eukaryotes and prokaryotes, in which they sequentially phosphorylate nucleosides before incorporation into a growing DNA strand [64]. Although poxvirus thymidine and thymidylate kinases similarly function in viral DNA replication, they are not highly conserved and are not required for replication of all poxviruses [58,65]. Furthermore, thymidine kinase has a proposed virulence role in VACV due to the drop in pathogenicity associated with thymidine kinase-negative viral recombinants [66]. F-box proteins are commonly found in eukaryotic genomes and contribute to the cellular ubiquitination system for protein degradation [67–69]. F-box-like proteins are also highly abundant virulence genes in CPV genomes, where they interact with host cell ubiquitin-proteasome components and lead to the degradation of important immunity-related proteins, such as nuclear factor kappa B (NF-kB) transcription factors [70–74]. Only one F-box domain-containing gene has been previously reported in an EPV, which is AMV254, a tryptophan repeat gene family protein in the AMEV genome [39]. The DlEPV putative F-box gene shows no similarity to AMV254 and differs from CPV F-box-like genes in that it does not contain the characteristic ankyrin repeats of poxvirus F-box-like genes [71]. In addition, the F-box domain of DLEV179 is located at the N-terminus, which is more similar to eukaryotic F-box proteins [75], rather than the C-terminal location common to viral F-box-like domains [71].

The DlEPV genome also contains three genes with protein domains not found in other viruses that may also be involved in virulence, such as a gamma-glutamyl transpeptidase domain (DLEV037), a type IV secretion system domain (DLEV099), and a thermostable hemolysin domain (DLEV172). The gamma-glutamyl transpeptidase (GGT) gene was first identified in DlEPV by Hashimoto and Lawrence [76]. A GGT has also been identified in the venom of *Aphidius ervi* parasitoid wasps, where it was shown to cause ovarian cell apoptosis in the aphid hosts of the wasp [77]. The DlEPV genes that encode the type IV secretion system (T4SS) and hemolysin domains could also be involved in host cell death, as these domains are used by many bacterial pathogens for cell membrane pore formation. T4SSs are used broadly by bacteria for the transfer of macromolecules, like DNA or proteins, to bacterial or eukaryotic cells [78,79]. Furthermore, hemolysins are toxins specifically used by bacterial pathogens, such as *Vibrio* species, to rupture blood cells of the infected host [80].

#### Additional putative virulence genes characterize the DlEPV genome

Due to the overall lack of poxvirus virulence gene conservation, we conducted a promoter sequence analysis to identify additional putative virulence genes in the DlEPV genome. Transcription of poxvirus genes is temporally regulated during infection, based on promoter sequence recognition by viral transcription factors that are specific to different stages of the replication cycle [56]. Viral genes transcribed soon after infection are known as early genes and include the virulence genes, which are expressed quickly to combat host defenses against infection [56,81,82]. CPV early genes contain a conserved upstream promoter sequence that is recognized by the early transcription factor packaged within virions [83–85]. Similarly, the promoter sequence TGAAAXXXXA is conserved among EPV early genes [39,41], so we searched the 100 bp upstream region of DlEPV ORFs without an assigned putative function for the EPV early promoter motif. We identified 34 additional putative virulence genes from this approach (Fig 1 and S1 Table). When combined with the 27 BRO genes and 6 virulence genes described above, a total of 67 putative virulence genes were identified in the DlEPV genome.

### Functional genomic analysis of DlEPV

We next used our annotation of the DlEPV genome to investigate the differential functionality of this virus in its two insect hosts. We have previously shown that DlEPV replicates in both wasp and fly tissue, but only flies are susceptible to the virulent effects of the virus [34]. In order to maintain a stable symbiosis, we hypothesized that DlEPV activity is altered within the wasp venom gland, such that replication is maximized and virulence is minimized. Conversely, we predicted that DlEPV replication and virulence activity follows a more standard poxvirus trajectory within the fly host. Because DlEPV shows no evidence of endogenization within the *D*. *longicaudata* genome, this variation in replication and virulence functions within the wasp would have to be achieved through a different mechanism than the genomic integration and reorganization feature of parasitoid EVEs. We therefore utilized RNA sequencing (RNA-seq) to examine whether differential viral gene expression is associated with the selective virulence demonstrated by DlEPV.

#### Identification of peak DlEPV expression for transcriptome sequencing

**Fig 4:**
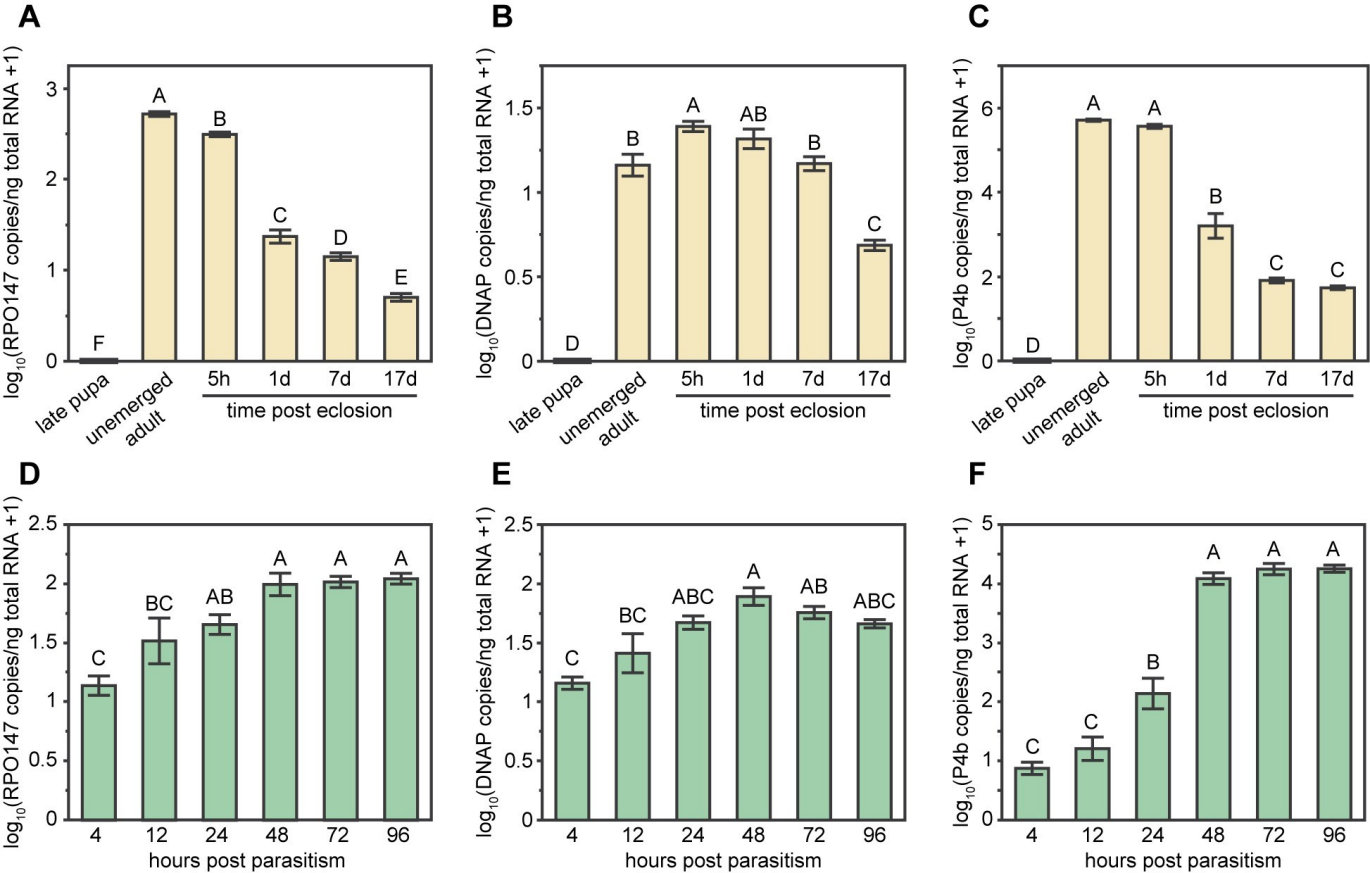
DlEPV gene expression profiles for selection of RNA-seq timepoints. Expression of DlEPV genes measured with RT-qPCR in (A-C) female wasp venom glands from late pupa to 17-day-old adult and (D-F) parasitized flies from 4–96 hours post parasitism (hpp). Profiled DlEPV genes encode (A,D) the 147kDa RNA polymerase subunit RPO147, (B,E) the DNA polymerase DNAP, and (C,F) the structural protein P4b. Each bar represents the mean log_10_ transformed cDNA copy number per ng total RNA averaged from 6 biological replicates. Error bars represent one standard error above and below the mean, and the letter(s) above each bar indicates statistically distinct mean values from Tukey’s HSD tests. Data in (A-C) were modified from Coffman *et al*. 2020 [34].

Stages of peak viral gene expression were first determined in both wasp and fly tissues to select for transcriptome sequencing. Our previously reported DlEPV expression profiles from wasp venom gland tissue indicated that viral gene expression peaked in female wasps at the time of the final molt into adulthood [34] (Fig 4A-4C). Here, we generated similar reverse transcription qPCR (RT-qPCR) profiles of whole flies throughout parasitism using the same 3 genes that were analyzed in venom gland tissue: the 147 kDa RNA polymerase subunit RPO147 (DLEV067), the DNA polymerase DNAP (DLEV168), and the P4b structural component (DLEV147). DlEPV gene expression rose steadily in flies 4–24 hours post parasitism (hpp) and plateaued at 48–96 hpp (RPO147 *F*_5,30_ = 12.29, *p* < 0.0001; DNAP *F*_5,30_ = 9.82, *p* < 0.0001; P4b *F*_5,30_ = 111.54, *p* < 0.0001) (Fig 4D-4F), which is congruent with prior qPCR quantification of viral genome copy growth in parasitized flies [34]. Using these data, we determined that the unemerged adult female wasp and the 72 hpp fly were comparable stages of maximum DlEPV expression activity.

#### Differential viral gene expression supports two DlEPV functional roles

**Fig 5:**
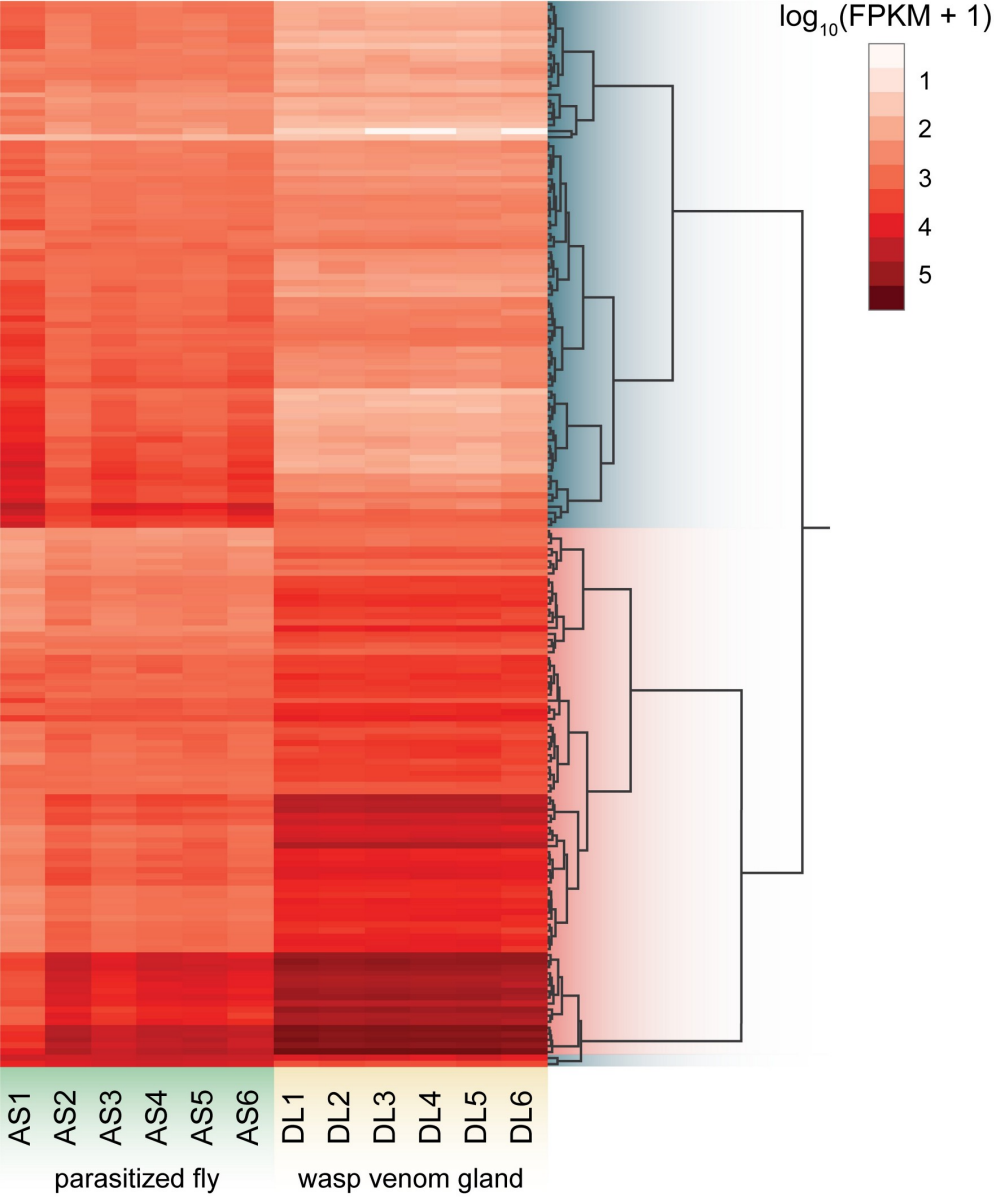
DlEPV shows widespread differential expression during replication in *D*. *longicaudata* wasps compared to *A*. *suspensa* flies. Heatmap showing significantly (FDR *q* < 0.05) differentially expressed DlEPV genes in wasp and fly tissue. Each row represents a DlEPV gene, and each column represents that gene’s expression in each of the 12 RNA samples. Expression is depicted as the log_10_ transformed FPKM value. Columns AS1-6 correspond to the 6 parasitized fly RNA replicates, and DL1-6 correspond to the 6 wasp venom gland replicates. Rows were clustered using the Ward method based on similarity in gene expression pattern across the 12 samples: DlEPV genes that were significantly downregulated in wasp tissue are highlighted in blue, and genes that were significantly upregulated in wasp tissue are highlighted in pink.

Total RNA was isolated from venom gland tissue of unemerged female wasps, as well as whole fly body tissue at 72 hpp (with wasp larva removed), including 6 biological replicates of each treatment. Because we had previously generated a transcriptome from unemerged wasp venom gland tissue [34], only 5 additional replicates were sequenced for this treatment. Paired-end sequencing followed by read quality filtering yielded an average 11.8 million read pairs per wasp sample and 8.9 million read pairs per fly sample. An average 38.9% of venom gland reads and 7.8% of parasitized fly reads aligned to the DlEPV genome. 91.2% (176 of 193) of DlEPV genes showed significant differential expression (FDR, *q* < 0.05) during virus replication in wasps and flies (S5 Table). Hierarchical clustering yielded two main groups of differentially expressed DlEPV genes: 86 genes were significantly upregulated, and 90 genes were significantly downregulated during virus replication in the wasp venom gland compared to the parasitized fly (Fig 5). Genes upregulated in wasp tissue displayed an average log_2_ fold change of 2.3, which is nearly a 5x greater level of expression in wasps compared to flies. Even more drastic were genes downregulated in wasp tissue, which had an average log_2_ fold change of 3.4, or >10x lower expression in wasps compared to flies (S5 Table). We then looked for differential expression patterns associated with the two main functional gene groups identified in the DLEPV genome: core replication genes and virulence genes. Remarkably, 82.2% (37 of 45) DlEPV core replication genes fell within the former cluster of genes upregulated in wasp tissue. The latter cluster of DlEPV genes downregulated in wasp tissue contained 79.1% (53 of 67) of DlEPV putative virulence genes, including 22 of the 27 BRO genes, 5 of the 6 virulence genes identified by sequence similarity, as well as 26 of the 34 additional virulence genes identified by their early promoter motif.

## Discussion

The genomic architecture of PDVs underpins many aspects of their associations with parasitoid wasps, including Mendelian inheritance of the proviral genome, the shift of virus replication control to the wasp, and restriction of replication to wasps and virulence to hosts [27]. We have previously shown that DlEPV broadly shares these features with PDVs as products of convergent evolution [34]. However, our work in this study has shown that DlEPV lacks the fundamental integration event that facilitated the evolution of these characteristics in PDV and other EVE systems. Our findings therefore raise intriguing questions regarding how features like vertical transmission, controlled virus replication, and selective virulence arose and are maintained in the DlEPV system. Furthermore, functional data presented here demonstrate that one of these features, partitioning of viral activity, is accomplished by DlEPV through a method not before observed for a beneficial virus.

### DlEPV represents an exogenous parasitoid virus

The presence of wasp genes surrounding multiple viral gene clusters is repeatedly observed with parasitoid EVE genome sequences [17–22]. Conversely, our assembly of the DlEPV genome into a single contig without bordering wasp DNA provides evidence that DlEPV is not integrated into the *D*. *longicaudata* genome and thus, is not an EVE. qPCR measurements of DlEPV genome copy number normalized by *D*. *longicaudata* genome copy number provide further support for the non-endogenous status of this virus, because they reveal that DlEPV is not consistently present in all wasp cells, which we would expect if DlEPV was an endogenous provirus. The notion that DlEPV is not integrated into the *D*. *longicaudata* genome is perhaps not surprising given the atypical replication strategy of other poxviruses. The family *Poxviridae* is unique among most other DNA viruses, as the poxvirus replication cycle is completed entirely within the cytoplasm of infected cells and does not require localization to the nucleus or integration of viral DNA into the host genome [38]. Nevertheless, the exogenous nature of the DlEPV genome contrasts starkly with the integrated and dispersed viral genome architecture of PDVs and other parasitoid EVEs [14,22].

Our DlEPV genome assembly is similar in total length to other poxvirus genomes, and our annotation of the DlEPV genome yielded the majority of conserved poxvirus core genes, indicating that we have successfully obtained the entire viral genome sequence. However, 4 of the 49 poxvirus core genes were not identified through sequence similarity searches. Given the absence of these genes in the *D*. *longicaudata* venom gland transcriptome, it is unlikely that these genes have integrated into the wasp genome. In addition, the high sequence divergence of identifiable DlEPV core genes displayed in our phylogeny suggests that the missing core genes still reside within the DlEPV genome but have diverged in sequence past the point of detection by our search methods. Furthermore, the similar level of sequence divergence in Yalta virus core genes, combined with the mutual absence of 2 core genes between the DlEPV and Yalta virus genomes support a lineage-specific divergence of these core genes. We can not rule out, however, that these genes may have instead been lost entirely from this poxvirus lineage and are not required for successful infection and replication within their respective hosts.

### DlEPV may have originated as a fly pathogen

Several EPVs with dipteran hosts have been described, but most representatives have been isolated from chironomid midges or mosquitoes and lack genetic sequence data [35]. Yalta virus is the first dipteran poxvirus isolated from the higher flies (suborder Brachycera), which also contains the tephritid fruit flies that serve as hosts for *D*. *longicaudata* wasps. The close relationship found between DlEPV and Yalta virus in this investigation supports the hypothesis that DlEPV arose from a fly pathogen. However, more taxonomic sampling of fly poxvirus genomes would be required to rule out the possibility that Yalta virus is instead a remnant parasitoid virus within a drosophilid host. How the DlEPV progenitor was acquired by the *D*. *longicaudata* lineage is unknown but could have occurred through a variety of events, since parasitoid wasps can come into contact with the pathogens of their hosts during development, as well as adulthood. For example, ascoviruses are pathogenic insect DNA viruses exclusively vectored to new lepidopteran hosts via contamination of adult parasitoid wasp ovipositors that are used to lay eggs within them [86]. The precise origin of most other parasitoid viruses and EVEs remains somewhat obscure due to limited taxonomic sampling of closely related insect DNA viruses, but many are suspected to be derived from pathogens of the parasitoids’ host insects [87]. The recent discoveries of hymenopteran and *Drosophila* poxviruses have allowed us to conduct a closer examination that suggests DlEPV originated as a pathogen from a host fly of the *D*. *longicaudata* ancestor. DlEPV thus provides more evidence for how viruses can be acquired by parasitoid wasps and lead to symbiogenesis events.

The genomic differences of DlEPV and Yalta virus compared to other sequenced EPVs suggest that insect poxviruses are more diverse than originally understood. The proximity of DlEPV and Yalta virus to CPVs in our unrooted phylogeny (Fig 2) makes the once clear divide between the EPV and CPV subfamilies appear more ambiguous. Other genomic features shared by DlEPV and Yalta virus differ from EPVs, such as their nucleotide base composition and gene content. Both DlEPV and Yalta virus contain higher than average GC content compared to other EPVs and therefore are more similar to CPVs, which can range widely in GC content from 25–65% [45]. The approximate 40% GC estimated for the *D*. *longicaudata* genome could also contribute to the elevated GC composition of DlEPV, in particular, due to the vertical transmission of this virus within an insect host to which other EPVs are not subjected. Additionally, apart from the core genes shared by all poxviruses, DlEPV and Yalta virus contain very few of the additional 50 genes shared by all EPVs [40,42]. One notably absent EPV-specific core gene from both DlEPV and Yalta virus genomes is that which encodes the protein spheroidin. This protein is not found in CPVs but is the main component of the characteristic EPV spheroid occlusion bodies, which are thought to protect EPV virions from environmental inactivation agents, such as UV light [88]. While the spheroidin gene may be divergent to a point beyond detection, a second scenario is that spheroidin is truly absent and not required for successful transmission of these viruses.

### Transcriptomic data support DlEPV functional dichotomy and genomic adaptations

The ability of DlEPV to replicate within both wasps and flies but only cause pathogenic effects during fly replication implies that DlEPV virulence is mitigated during virus replication in wasps. This strategy would promote a more stable association between virus and wasp, but as DlEPV is not endogenous, the viral genome integration and dispersion observed in other parasitoid viruses fails to explain how DlEPV completes nonpathogenic replication within wasp tissue. We looked at differential viral gene expression during replication in wasps and flies as an alternative mechanism that might corroborate the selective virulence phenotype of DlEPV. Our findings demonstrate that DlEPV transcriptional activity varies largely during replication in wasps compared to flies, supporting a promotion of virus replication and inhibition of virulence in wasp tissue. These distinct expression patterns are correlated to the different predicted roles of the virus in its two hosts: maximum virus replication in wasp tissue produces an abundance of virions for injection into hosts during oviposition, and restriction of virulence to fly tissue manipulates the host physiology for successful parasitism by the developing wasp. Of note, putative virulence genes, such as the BRO genes, were expressed at extremely low levels compared to other DlEPV genes during virus replication in the venom gland, and they represented many of the most differentially expressed viral genes in wasps compared to flies. We hypothesize that regulatory mechanisms exist within *D*. *longicaudata* that suppress BRO and other virulence gene expression during virus replication in the venom gland to deter viral pathogenicity. Possible measures of DlEPV control such as this warrant future study, because they differ from what is observed in PDV systems. The differential DlEPV gene expression reported here thus represents convergent evolution with endogenous parasitoid viruses to maintain a separation of viral function that aligns with parasitoid wasp survival and fitness.

Results from our transcriptomes also hint that DlEPV genomic features, such as the BRO gene expansion, are adaptive to symbiotic life. The DlEPV BRO genes are far more extensive in copy number than observed in other EPVs, likely due to a large tandem duplication in the DlEPV genome center. In addition, the majority of DlEPV BRO genes demonstrated upregulation in fly tissue, supporting their involvement in virulence within the fly hosts of *D*. *longicaudata* wasps. Poxviruses have experimentally been shown to undergo rapid, tandem virulence gene duplications as a means of adaptation [89,90]. Therefore, tandem duplication of the BRO genes may be adaptive to the success of DlEPV, or by extension, the success of the developing wasp that is also fighting for survival within the fly host. Similar to DlEPV, PDV genomes contain large gene families with members that function as host virulence factors, such as the protein tyrosine phosphatases (PTPs) [91]. Several PTP members arose by tandem duplication events, and some also show evidence for positive selection [92]. The gene duplications in DlEPV and PDV genomes may therefore represent similar adaptations to respective hosts due to the shared selective pressures accompanied by their associations with parasitoid wasps.

### DlEPV is a true mutualistic viral symbiont of parasitoid wasps

Heritable associations between insects and beneficial microbes are often highly complex, due to the unconventional evolutionary forces that act on host-associated microbes [93]. Vertically transmitted bacterial symbionts that are completely restricted to live within insect hosts often exhibit extreme genome degradation compared to their free-living relatives [94,95]. Even though this genomic decay causes the bacteria to become dependent on their host for survival, the symbionts still maintain an exogenous genome that replicates and evolves separately from the host genome [94,95]. PDVs are similar to many of these bacteria in that they provide essential functions for their parasitoid wasp hosts. However, PDVs are less commonly considered to be true symbionts, because they do not contain a replicative genome external to the wasp genome [96]. Until now, genetic characterization of heritable parasitoid viruses has challenged the very notion of a ‘viral symbiont’ given the shared endogenous nature of those currently described. Furthermore, known examples of heritable viruses that are not endogenous, such as DpTV and DcPV, remain to be definitively demonstrated as mutualistic. DlEPV is thus an unprecedented example of a virus that fully meets the requirements of a heritable mutualistic symbiont, including an exogenous genome and a beneficial function within *D*. *longicaudata* wasps. As the first genuine mutualistic viral symbiont of parasitoid wasps to be characterized, DlEPV shows promise as a tractable system from which to gain valuable knowledge on the viral side of microbial mutualism in insects.

## Materials and methods

### Viral genome sequencing and assembly

*D*. *longicaudata* wasps and *A*. *suspensa* flies were reared as previously described [97]. Dissected venom glands from six *D*. *longicaudata* adult female wasps were pooled into one sample to enrich for DlEPV DNA, followed by phenol:chloroform DNA extraction as reported previously [34]. The resulting DNA was subjected to both Pacific BioSciences (PacBio) and Illumina technologies to sequence the DlEPV genome. 7.5 μg of venom gland DNA was used to make a 10 kb insert size library using PacBio standard SMRT library construction chemistry. The PacBio library was sequenced using a 120 min movie on one SMRT cell. PacBio data were analyzed using the smrtanalysis-2.2.0 Amazon Machine Image hosted on Amazon Web Services. 150,283 PacBio reads were filtered to retain 78,011 reads with a minimum read score of 0.8 and length of 100 bp. 615 long reads (>6 kb in length) were pre-assembled and error-corrected by aligning short reads (>500 bp) to the longer reads and taking the consensus with HGAP v3.0 [98]. Following this, 522 long error-corrected reads with an N50 of 9,071 bp were assembled into 19 unitigs to form a draft assembly using the Celera Assembler [99]. All reads were aligned to the assembly to give coverage reports and perform polishing with Quiver from SMRT Analysis v2.2. Each unitig was split into 3 kb pieces and analyzed with blastx against the NCBI non-redundant (nr) protein sequence database (downloaded in September 2014). 7 unitigs of putative EPV origin were selected based upon BLAST results and unitig depth of sequence coverage. These unitigs were compared to each other using blastn, which revealed sets that were almost identical and completely nested within each other, and may have been split apart during assembly due to differing numbers of short repeat sequences in each assembled unitig. The nested unitigs were excluded to retain the longest version of each sequence, resulting in three final unitigs. These unitigs contained areas of overlap ranging from 5–10 kb in length, which were assembled manually to form the full DlEPV genome.

Illumina-compatible library construction was performed using 1 μg of venom gland DNA and the standard protocol with Kapa Biosystems DNA library preparation chemistry. The library was sequenced with 8.6 million 75 bp paired-end reads on an Illumina MiSeq instrument at the Georgia Genomics and Bioinformatics Core (GGBC). Reads were filtered with the fastx toolkit (hannonlab.cshl.edu/fastx_toolkit/) to retain reads with >90% of bases with a PHRED score of 30 or higher for both reads in a pair. 6,243,436 read pairs were mapped against the DlEPV genome assembly with bowtie2 v2.2.4 [100] to correct any potential errors that arose from PacBio sequencing. Variant SNPs, insertions, and deletions present in the Illumina short read alignment were identified with SAMtools v1.0 [101], as well as through manual inspection. 140 total corrections were made to the reference genome.

### DlEPV genome annotation

ORFs with a methionine start codon and a length of at least 50 amino acids were predicted using a combination of Prokka v1.6 [102] and Artemis v16 [103]. ORFs with highly repetitive amino acid sequences were manually discarded as illegitimate proteins. The remaining ORFs were subjected to blastp protein searches against the nr database (downloaded in September 2019), as well as custom BLAST databases composed of poxvirus proteins (taxid: 10240) or strictly EPV proteins (taxid: 10284). Conserved protein domain searches were also conducted against the Pfam database using hmmsearch from HMMER v3.1b1 [104]. An *e*-value cutoff of 0.001 for blastp searches and 0.01 for Pfam searches were used for the bulk of viral gene annotations. These combined searches provided putative functions for 88 of the 193 identified DlEPV ORFs, including 44 of the 49 poxvirus core genes.

To find distant homologs to the poxvirus core genes missed by initial blastp and Pfam searches, we looked for possible matches to the core genes that were beyond our original *e*-value cutoffs but found no additional hits despite these relaxed blastp and Pfam search parameters. We then scoured the DlEPV gene set using core gene hidden Markov model (HMM) searches. HMMs were constructed for each poxvirus core gene by aligning amino acid sequences from available EPV and VACV orthologs with MAFFT v7.215 using the—auto alignment setting [105] and hmmbuild from HMMER. Each HMM was then queried against all DlEPV protein sequences with hmmscan. Only one additional core gene, the L5R entry/fusion membrane protein (DLEV060), was identified from this approach. We also queried our HMMs against intergenic regions of the DlEPV genome to identify core genes that may have been pseudogenized but found no hits from this approach.

A *de novo* transcriptome assembly was generated from previously published venom gland RNA-seq reads (accession GSE144541) to check if the four remaining core genes had integrated into the *D*. *longicaudata* genome. First, bowtie2 v2.2.4 was used to map quality filtered reads from the venom gland transcriptome to the DlEPV genome. Reads that failed to map to the reference genome were collected and fed as input for *de novo* transcript assembly using Trinity v2.0.6 [106]. A BLAST nucleotide database was created from the resulting assembly, and the missing core genes were queried against it with tblastn. We then queried the HMMs of the missing genes against the translated transcriptome assembly with HMMER but found no significant hits from either approach.

### Comparative genomic analyses

We used publicly available annotations to calculate genome metrics for the majority of poxvirus genomes featured in this study: ACEV (accession NC_023426), AHEV (NC_021247), AMEV (NC_002520), CBEV (NC_021248), CREV (NC_021249), MSEV (NC_001993), MySEV (NC_021246), Yalta virus (MT364305), VACV (NC_006998), ORFV (NC_005336), MOCV (NC_001731), FWPV (NC_002188), CRV (NC_008030), and SGPV (NC_027707). However, the partial LHEV genome (NC_040577) required re-annotation for use in our analyses. We used Prokka v1.13 to call ORFs within the 46 kb LHEV genome segment and identified 53 total ORFs (S6 Table). BLAST searches against poxvirus and EPV protein databases yielded 18 LHEV ORFs that showed similarity to poxvirus core genes. There were two instances in which two adjacent ORFs had sequence similarity to opposite ends of the same core gene. In both cases, the two ORFs had the same strand orientation and were separated by a single frame shift. Therefore, we assumed the original core gene was incorrectly split into two ORFs due to a single nucleotide sequence error. A total of 16 unique core genes were thus identified from the partial LHEV genome.

Nucleotide composition (% GC) was estimated for the *D*. *longicaudata* and *A*. *suspensa* genomes using RNA-seq transcriptomes that were generated as described below. For each species, Trinity was used to construct a *de novo* assembly from RNA reads that failed to map to the DlEPV genome combined for all 6 RNA replicate samples per insect. GC content was then calculated from the resulting assemblies.

To generate the poxvirus core gene phylogeny, amino acid sequences for EPV and CPV orthologs of the 16 core genes found in LHEV (S3 Table) were aligned with MAFFT—auto, concatenated using Geneious Prime 2019.2.3 (https://www.geneious.com), and trimmed of alignment positions in which >50% of taxa contained a gap using trimAl v1.4.1 [107]. The ML phylogenetic tree was generated using RAxML v8.2.11 [108], in which the Gamma model of rate heterogeneity and the LG amino acid substitution matrix were utilized.

### RNA isolation and RT-qPCR estimation of viral gene expression

DlEPV gene expression in host flies during parasitism was measured by offering third instar fly larvae to 7-day-old adult wasps that had no prior oviposition experience for 2 h. Resulting flies containing a single laid wasp egg (i.e. those with one oviposition scar) were collected at 4–96 hpp. Flies were kept in standard rearing conditions until each sampling time point. Whole fly samples were collected in a guanidine hydrochloride lysis buffer consisting of 4.9M guanidine hydrochloride, 2% sarkosyl, 50 mM Tris-Cl (pH 7.6), and 10 mM EDTA. Total RNA was isolated using phenol:chloroform, followed by DNase treatment with the TURBO DNA-*free* Kit (Ambion), and elution in 30 μL water. First-strand cDNA was synthesized with 1,000 ng fly RNA according to the Superscript III reverse transcriptase protocol (Invitrogen) using oligo(dT) primers. qPCR reactions were performed as described previously for wasp venom gland DlEPV expression profiling [34]. JMP Pro 14 was used for statistical analysis of RT-qPCR data. One-way ANOVA was used to test for differences in means of biological replicates, and Tukey’s HSD was used for multiple comparison tests. Copy numbers were log_10_ transformed prior to statistical analysis.

### Transcriptome sequencing and analysis

Unemerged wasp venom glands were collected in triplicate as was done for our initial venom gland transcriptome to obtain 5 additional venom gland samples for a total of 6 biological replicates. Singly-scarred fly pupae were collected 72 hpp by first removing the developing wasp larva by dissection in PBS. A total of 6 flies were collected in this manner with each specimen representing one biological replicate. Total RNA for sequencing was extracted using the RNeasy Mini Kit (QIAGEN) with on-column DNase digestion, followed by a secondary DNase treatment using the TURBO DNA-*free* Kit (Ambion) after RNA isolation. Illumina-compatible stranded RNA libraries for the 12 samples were constructed at the GGBC with the Kapa Biosystems RNA library preparation chemistry using 3 μg RNA from each fly sample and 1μg RNA from each venom gland sample. Libraries were sequenced using an Illumina NextSeq instrument at the GGBC, which generated an average of 18.5 million and 14.2 million 75 bp paired-end reads for each wasp and fly sample, respectively. Reads were quality filtered using the fastx toolkit as described before with the Illumina DNA sequencing. Quality reads for each sample were separately mapped to the DlEPV reference genome using bowtie2 v2.2.4. Cuffquant from Cufflinks v2.2.1 was used to calculate the average fragments per kilobase of transcript per million mapped reads (FPKM) values for each DlEPV ORF in both wasp and fly tissues, and Cuffdiff was used to test for differential expression between the two treatments [109]. Differential expression for a gene was considered significant for FDR-adjusted *q*-values < 0.05 [110]. Hierarchical clustering of the significantly differentially expressed DlEPV genes was performed with JMP Pro 14.

## Supporting information

S1 Table Annotated ORFs in the DlEPV genome. (PDF)

S2 Table Sequenced EPV genome features. (PDF)

S3 Table Poxvirus core gene homologs in EPV and CPV genomes. The 49 poxvirus core genes are shown with corresponding locus tags for homologous ORFs in EPV and CPV genomes. The 16 core genes used to build the phylogeny in Fig 2 are highlighted in yellow.
(PDF)

S4 Table BRO genes in EPV genomes. BRO genes are defined as those with a Bro-N protein domain. Protein domains were identified using hmmsearch to query genes from each genome against the Pfam database. A maximum *e*-value cutoff of 0.05 was used to isolate significant domain matches.
(PDF)

S5 Table Expression of DlEPV genes in wasp venom gland (DL) and parasitized fly (AS) samples. Genes are grouped by their putative function based on similarity to other poxvirus genes. Genes of putative virulence function are subdivided between those with a Bro-N domain (Virulence: BRO Genes), those that were identified by sequence similarity to known virulence genes (Virulence: Homology), and those that have a conserved EPV early gene promoter motif and no other assigned function (Virulence: Early Promoter). Genes with an asterisk indicate those that demonstrated significant differential expression between the two treatments (*q* < 0.05).
(PDF)

S6 Table Re-annotation of the LHEV genome segment. Feature table of LHEV ORFs including the 11 ORFs previously annotated by Viljakainen *et al*. 2018 [43] (accession NC_040577).
(PDF)

S1 Fig Normalized DlEPV abundance in *D*. *longicaudata* wasps. DlEPV copy number relative to *D*. *longicaudata* copy number was estimated with qPCR for (A) adult female wasp reproductive tissues, and (B) female and male whole wasps in pupal-adult developmental stages. Venom glands and ovaries from adult females were pooled in triplicate for each biological replicate. DlEPV genome copy number was estimated using the poly(A) polymerase small subunit gene (polyAPol, DLEV167), and *D*. *longicaudata* copy number with the elongation factor alpha gene (EF1a). qPCR was performed as done previously [34]. The y-axes indicate the log_10_ fold change of total DlEPV genome copy number over total *D*. *longicaudata* genome copy number. Permanent integration of the DlEPV genome into the *D*. *longicaudata* genome would result in a ratio of virus to wasp copy number that is ≥ 1 for all wasp tissues, developmental stages, and sexes, which is equivalent to a log_10_ abundance fold change of 0. Negative log_10_ abundance fold change values indicate samples in which the virus to wasp copy number ratio was < 1. Each bar represents the average relative DlEPV copy number across 6 biological replicates, and error bars represent one standard error above and below the mean.
(PDF)

S2 Fig Additional poxvirus core gene phylogenies. (A) Maximum likelihood (ML) phylogenetic tree built from a concatenated multiple sequence alignment of the 44 core genes shared by all EPV complete genomes. Methods were the same as used to build the phylogeny in Fig 2. Node support (%) was inferred with 1,000 bootstrap iterations. (B) Bayesian inference phylogeny of the concatenated 16 core genes from Fig 2 built using PhyloBayes-MPI v20161021 with the CAT-GTR substitution model [111]. Node support in panel B is labeled with the consensus posterior probability from two independent Markov chain Monte Carlo simulations that ran for 10,000 cycles each with a 1,000 cycle burn-in. (C) ML phylogeny built from a concatenated alignment of the 10 poxvirus core genes shared by all poxviruses and sister group Asfarviridae members African swine fever virus (ASFV, NC_001659) and Kaumoebavirus (NC_034249). Core genes and respective ASFV and Kaumoebavirus accession numbers used to create the phylogeny in panel C include the DNA polymerase E9L (AAA65319.1, ARA71927.1), RNA helicase I8R (AAA65302.1, ARA71975.1), RPO147 J6R (AAA65328.1, ARA71945.1 and ARA71948.1), mRNA-capping enzyme large subunit D1R (AAA65330.1, ARA71993.1), NTPase, DNA primase D5R (AAA65301.1, ARA71965.1), viral early transcription factor (VETF) small subunit D6R (AAA65335.1, ARA72203.1), VETF large subunit A7L (AAA65318.1, ARA71923.1), ATPase NPH1 D11R (AAA65350.1, ARA72259.1), RPO132 A24R (AAA65283.1, ARA72182.1), and ATPase/DNA-packaging protein A32L (AAA65308.1, ARA72015.1). Tree building methods were the same as done for other ML trees.
(PDF)
