# Supplementary material for: Genomic analysis reveals an exogenous viral symbiont with dual functionality in parasitoid wasps and their hosts
Source: PLoS Pathog. 2020 Nov 30;16(11):e1009069. doi: 10.1371/journal.ppat.1009069 (PMC7728225; doi:10.1371/journal.ppat.1009069)
Supplement: S1 Table — (PDF) [file ppat.1009069.s001.pdf]

S1 Table. Annotated ORFs in the DlEPV genome.

Locus Tag	Start	End	Size (aa)	Gene ID	Best BLAST Hit				Pfam Domain(s)	VACV Core Gene	Yalta virus Ortholog(s)*	Early Promoter	Putative Function
					Accession	Source Species	Size (aa)	Identity (%)					
DLEV001/193	4080	3826	85										Unknown
DLEV002/192	5545	5384	54										Unknown
DLEV003/191	6463	5960	168	BRO protein					Bro-N			Yes	Virulence: BRO Genes
DLEV004/190	7243	6851	131	unknown protein	YP_001649041.1	Helicoverpa armigera granulovirus	684	37					Unknown
DLEV005/189	8847	8389	153										Unknown
DLEV006/188	9621	9208	138									Yes	Virulence: Early Promoter
DLEV007/187	9692	10117	142	deoxyuridine 5'-triphosphate nucleotidohydrolase (dUTPase)	XP_012261123.1	*Athalia rosae*	151	64	dUTPase		Yalta_053		Replication
DLEV008/186	14962	14165	266									Yes	Virulence: Early Promoter
DLEV009/185	17646	17191	152									Yes	Virulence: Early Promoter
DLEV010	17946	17710	79										Unknown
DLEV011	18885	18370	172									Yes	Virulence: Early Promoter
DLEV012	19683	19330	118	BRO protein					Bro-N			Yes	Virulence: BRO Genes
DLEV013	20800	20048	251						HALZ				Unknown
DLEV014	21247	20822	142	BRO protein	NP_064959.1	Amsacta moorei entomopoxvirus	360	30	Bro-N				Virulence: BRO Genes
DLEV015	21961	21248	238									Yes	Virulence: Early Promoter
DLEV016	22072	22818	249	unknown protein	XP_004224708.1	*Plasmodium cynomolgi* strain B	1017	32	DUF4638			Yes	Virulence: Early Promoter
DLEV017	23517	22813	235	unknown protein	SPJ09464.1	*Plasmodium* sp. DRC-Itaito	781	33	Gal11_ABD1				Unknown
DLEV018	23561	23788	76										Unknown
DLEV019	24984	23797	396	reverse transcriptase	YP_008003836.1	Adoxophyes honmai entomopoxvirus	283	30	RVT_1		Yalta_167	Yes	Transcription/RNA Modification
DLEV020	28703	27114	530	nucleoside triphosphatase/helicase 1	ARF10049.1	Indivirus ILV1	487	28	IBR		Yalta_006	Yes	Transcription/RNA Modification
DLEV021	28779	30068	430	serine/threonine protein kinase	AAO31700.1	Orf virus strain D1701	497	26	Pox_ser-thr_kin	F10L	Yalta_093		Structure/Morphogenesis
DLEV022	31749	31096	218								Yalta_092		Unknown
DLEV023	31778	32713	312								Yalta_089		Unknown
DLEV024	32714	34705	664	viral early transcription factor small subunit (VETFS)	NP_064956.1	Amsacta moorei entomopoxvirus	670	37	SNF2_N	D6R	Yalta_024		Transcription/RNA Modification
DLEV025	36868	36287	194						TraC			Yes	Virulence: Early Promoter

Locus Tag	Start	End	Size (aa)	Gene ID	Best BLAST Hit				Pfam Domain(s)	VACV Core Gene	Yalta virus Ortholog(s)*	Early Promoter	Putative Function
					Accession	Source Species	Size (aa)	Identity (%)					
DLEV026	36906	38768	621	metalloprotease	YP_009268770.1	Pteropox virus	593	25		G1L	Yalta_131		Structure/Morphogenesis
DLEV027	38769	39014	82										Unknown
DLEV028	42741	40258	828	ribonucleoside-diphosphate reductase large subunit (RNRL)	OJU81738.1	*Chlamydia sp.* 32-24	771	30	Ribonuc_red_lgC		Yalta_121	Yes	Replication
DLEV029	44772	44047	242	RNA polymerase 18kDa subunit (RPO18)	YP_009001517.1	Anomala cuprea entomopoxvirus	180	31	Pox_RNA_pol	D7R	Yalta_120	Yes	Transcription/RNA Modification
DLEV030	45089	44787	101										Unknown
DLEV031	46149	45148	334	ribonucleoside-diphosphate reductase small subunit (RNRS)	AEO98243.1	Emiliania huxleyi virus 203	325	28	Ribonuc_red_sm		Yalta_116	Yes	Replication
DLEV032	47451	46963	163										Unknown
DLEV033	49666	48401	422								Yalta_142		Unknown
DLEV034	49695	50609	305	mRNA capping enzyme small subunit	NP_048195.1	Melanoplus sanguinipes entomopoxvirus	267	33	Pox_mRNA-cap	D12L	Yalta_143		Transcription/RNA Modification
DLEV035	51032	50598	145	late membrane protein	YP_008004034.1	Adoxophyes honmai entomopoxvirus	139	38	Pox_G9-A16	J5L	Yalta_056		Structure/Morphogenesis
DLEV036	51046	51801	252	ssDNA/dsDNA binding protein					Pox_VP8_L4R	L4R	Yalta_059		Structure/Morphogenesis
DLEV037	54427	52940	496	gamma-glutamyl transpeptidase	XP_018319317.1	*Agrilus planipennis*	613	27	G_glu_transpept			Yes	Virulence: Homology
DLEV038	54439	55401	321	alpha/beta fold hydrolase	WP_052830421.1	*Gynuella sunshinyii*	325	29	Hydrolase_4		Yalta_165		Unknown
DLEV039	58130	55491	880	DNA primase	YP_008003627.1	Mythimna separata entomopoxvirus	730	29	D5_N	D5R	Yalta_152		Replication
DLEV040	58168	58845	226	S-S bond formation pathway protein substrate	ARE67656.1	Shearwaterpox virus	213	31	L1R_F9L	F9L	Yalta_151		Structure/Morphogenesis
DLEV041	58847	59077	77										Unknown
DLEV042	60506	59160	449	virion core cysteine protease	YP_008004293.1	Choristoneura biennis entomopoxvirus	459	30	Peptidase_C57	I7L	Yalta_147		Structure/Morphogenesis
DLEV043	60524	61945	474										Unknown
DLEV044	62705	63949	415	FEN1-like nuclease	YP_008003980.1	Adoxophyes honmai entomopoxvirus	414	24		G5R	Yalta_145	Yes	Structure/Morphogenesis
DLEV045	66551	65640	304	adenosine triphosphatase/DNA packaging protein	YP_008003707.1	Mythimna separata entomopoxvirus	238	32	Pox_A32	A32L	Yalta_094		Structure/Morphogenesis
DLEV046	68862	67771	364	myristylated membrane protein	NP_048161.1	Melanoplus sanguinipes entomopoxvirus	380	35	Pox_G9-A16	A16L	Yalta_134		Structure/Morphogenesis
DLEV047	69540	68863	226	uracil DNA glycosylase	OUU96308.1	*Flavobacteriales* bacterium TMED84	220	46	UDG	D4R		Yes	Replication

Locus Tag	Start	End	Size (aa)	Gene ID	Best BLAST Hit				Pfam Domain(s)	VACV Core Gene	Yalta virus Ortholog(s)*	Early Promoter	Putative Function
					Accession	Source Species	Size (aa)	Identity (%)					
DLEV048	70287	69913	125										Unknown
DLEV049	70664	70314	117	IMV entry/fusion membrane protein	YP_008004356.1	Choristoneura biennis entomopoxvirus	112	29	Pox_A21	A21L	Yalta_138		Structure/Morphogenesis
DLEV050	71837	70950	296									Yes	Virulence: Early Promoter
DLEV051	71889	73685	599	unknown protein	YP_008004224.1	Choristoneura biennis entomopoxvirus	438	28			Yalta_135		Unknown
DLEV052	74098	73709	130									Yes	Virulence: Early Promoter
DLEV053	76814	75423	464								Yalta_144	Yes	Virulence: Early Promoter
DLEV054	77163	76795	123										Unknown
DLEV055	77168	77728	187	RNA polymerase 19kDa subunit (RPO19)	NP_064948.1	Amsacta moorei entomopoxvirus	237	43		A5R	Yalta_133	Yes	Transcription/RNA Modification
DLEV056	78513	78094	140									Yes	Virulence: Early Promoter
DLEV057	78988	78593	132						PIG-P				Unknown
DLEV058	80923	79784	380	RNA helicase	EYB84565.1	*Ancylostoma ceylanicum*	384	59	DEAD, Helicase_C			Yes	Transcription/RNA Modification
DLEV059	82820	81738	361	viral intermediate transcription factor 3 large subunit (VITF3L)	YP_008003912.1	Adoxophyes honmai entomopoxvirus	353	22		A23R	Yalta_095		Transcription/RNA Modification
DLEV060	82832	83284	151	IMV entry/fusion membrane protein	YP_009480602.1	Sea otter poxvirus	133	28		L5R	Yalta_096		Structure/Morphogenesis
DLEV061	85113	84076	346	internal virion protein	YP_009001617.1	Anomala cuprea entomopoxvirus	346	28	Pox_L3_FP4	L3L	Yalta_060		Transcription/RNA Modification
DLEV062	85130	86026	299	myristylated membrane protein	YP_008003859.1	Adoxophyes honmai entomopoxvirus	336	27	Pox_G9-A16	G9R	Yalta_067		Structure/Morphogenesis
DLEV063	87113	86793	107	viral membrane-associated, early morphogenesis protein	NP_064943.1	Amsacta moorei entomopoxvirus	81	33		A9L	Yalta_141	Yes	Structure/Morphogenesis
DLEV064	87119	89479	787	viral early transcription factor large subunit (VETFL)	NP_048134.1	Melanoplus sanguinipes entomopoxvirus	760	25	Pox_VERT_large	A7L	Yalta_140	Yes	Transcription/RNA Modification
DLEV065	89488	90594	369								Yalta_139	Yes	Virulence: Early Promoter
DLEV066	90572	91159	196	entry/fusion membrane protein	YP_009001651.1	Anomala cuprea entomopoxvirus	201	42	Pox_LP_H2	H2R	Yalta_125	Yes	Structure/Morphogenesis
DLEV067	95846	91929	1306	RNA polymerase 147kDa subunit (RPO147)	AAN01131.1	Anomala cuprea entomopoxvirus	1318	30	RNA_pol_Rpb1_1, RNA_pol_Rpb1_2, RNA_pol_Rpb1_3, RNA_pol_Rpb1_4, RNA_pol_Rpb1_5	J6R	Yalta_105	Yes	Transcription/RNA Modification
DLEV068	97178	96780	133								Yalta_087		Unknown

Locus Tag	Start	End	Size (aa)	Gene ID	Best BLAST Hit				Pfam Domain(s)	VACV Core Gene	Yalta virus Ortholog(s)*	Early Promoter	Putative Function
					Accession	Source Species	Size (aa)	Identity (%)					
DLEV069	97853	97218	212										Unknown
DLEV070	97858	98571	238										Unknown
DLEV071	98708	98442	89										Unknown
DLEV072	100483	98993	497	NAD-dependent DNA ligase	AYV79438.1	Faunusvirus sp.	640	31	DNA_ligase_OB		Yalta_084	Yes	Replication
DLEV073	102995	102645	117									Yes	Virulence: Early Promoter
DLEV074	103031	103531	167								Yalta_080	Yes	Virulence: Early Promoter
DLEV075	104764	103541	408								Yalta_073		Unknown
DLEV076	105366	104824	181	oligoribonuclease	XP_018347894.1	*Trachymyrmex septentrionalis*	180	48	RNase_T				Transcription/RNA Modification
DLEV077	105404	105892	163										Unknown
DLEV078	107981	106539	481	DNA helicase	NP_073495.1	Yaba-like disease virus	479	29	ResIII, Helicase_C	A18R	Yalta_075		Transcription/RNA Modification
DLEV079	108344	107991	118						CRA				Unknown
DLEV080	108700	108359	114						DUF4094, RILP				Unknown
DLEV081	108707	110704	666	nucleoside triphosphatase I	NP_048124.1	Melanoplus sanguinipes entomopoxvirus	647	33	NPHI_C, Helicase_C, SNF2_N	D11R	Yalta_079		Transcription/RNA Modification
DLEV082	111550	111146	135						Bro-N			Yes	Virulence: BRO Genes
DLEV083	112971	112156	272	BRO protein	YP_009046672.1	Armadillidium vulgare iridescent virus	231	32	Bro-N, T5orf172			Yes	Virulence: BRO Genes
DLEV084	114229	113669	187	BRO protein	YP_003517810.1	Lymantria xylina nucleopolyhedrovirus	249	37	Bro-N, T5orf172			Yes	Virulence: BRO Genes
DLEV085	114677	114324	118										Unknown
DLEV086	115026	114751	92									Yes	Virulence: Early Promoter
DLEV087	115835	115125	237										Unknown
DLEV088	116526	115861	222										Unknown
DLEV089	117212	116529	228										Unknown
DLEV090	117716	117384	111										Unknown
DLEV091	119106	118663	148									Yes	Virulence: Early Promoter
DLEV092	119955	119158	266	BRO protein					Bro-N			Yes	Virulence: BRO Genes
DLEV093	121604	121050	185									Yes	Virulence: Early Promoter
DLEV094	122914	122243	224	BRO protein					Bro-N			Yes	Virulence: BRO Genes
DLEV095	123585	123016	190						CC2-LZ			Yes	Virulence: Early Promoter

Locus Tag	Start	End	Size (aa)	Gene ID	Best BLAST Hit Accession	Source Species	Size (aa)	Identity (%)	Pfam Domain(s)	VACV Core Gene	Yalta virus Ortholog(s)*	Early Promoter	Putative Function
DLEV096	124627	123779	283	BRO protein	AJP09121.1	Heliothis virescens ascovirus 3f	158	37	Bro-N			Yes	Virulence: BRO Genes
DLEV097	126880	126116	255	BRO protein					Bro-N			Yes	Virulence: BRO Genes
DLEV098	128318	127533	262	BRO protein	AOL56971.1	Chrysodeixis includens nucleopolyhedrovirus	499	32	Bro-N			Yes	Virulence: BRO Genes
DLEV099	129352	128576	259						T4SS			Yes	Virulence: Homology
DLEV100	130617	129940	226	BRO protein	AXN77333.1	Heliothis virescens ascovirus 3i	343	27	Bro-N, T5orf172			Yes	Virulence: BRO Genes
DLEV101	131376	130726	217	BRO protein	WP_069467284.1	*Streptomyces rubidus*	316	57	Bro-N			Yes	Virulence: BRO Genes
DLEV102	131920	131444	159										Unknown
DLEV103	132245	131940	102	BRO protein	YP_009121837.1	Spodoptera frugiperda granulovirus	252	40	Bro-N			Yes	Virulence: BRO Genes
DLEV104	133579	133037	181						Uds1				Unknown
DLEV105	134350	133649	234	BRO protein	BBB16628.1	Heliothis virescens ascovirus 3j	496	34	Bro-N, ACCA			Yes	Virulence: BRO Genes
DLEV106	135696	135340	119									Yes	Virulence: Early Promoter
DLEV107	136156	135788	123	BRO protein					Bro-N				Virulence: BRO Genes
DLEV108	137198	136257	314						MIEAP			Yes	Virulence: Early Promoter
DLEV109	137907	137236	224	BRO protein	YP_009121837.1	Spodoptera frugiperda granulovirus	252	31	Bro-N			Yes	Virulence: BRO Genes
DLEV110	140837	139749	363	viral membrane formation protein	NP_064920.1	Amsacta moorei entomopoxvirus	320	28	Phage_int_SAM_6	A11R	Yalta_072		Structure/Morphogenesis
DLEV111	140868	144182	1105	P4a precursor	QGM48880.1	Magpiepox virus	893	30		A10L	Yalta_071		Structure/Morphogenesis
DLEV112	145744	144188	519										Unknown
DLEV113	146491	147072	194	Holliday junction resolvase	YP_008003995.1	Adoxophyes honmai entomopoxvirus	159	29	Pox_A22	A22R	Yalta_069		Replication
DLEV114	149654	147105	850	unknown protein	RUS86699.1	*Elysia chlorotica*	333	27	Phage_XkdX, FlaC_arch, DUF2939		Yalta_068		Unknown
DLEV115	150917	149661	419						RnlB_antitoxin, Phage_T7_tail, CALCOCO1		Yalta_068		Unknown
DLEV116	152432	150933	500								Yalta_068		Unknown
DLEV117	153053	153838	262										Unknown
DLEV118	154369	155607	413	BRO protein	YP_008004169.1	Choristoneura biennis entomopoxvirus	347	30	Bro-N, DUF3627			Yes	Virulence: BRO Genes
DLEV119	156416	156027	130	BRO protein					Bro-N			Yes	Virulence: BRO Genes

Locus Tag	Start	End	Size (aa)	Gene ID	Best BLAST Hit Accession	Source Species	Size (aa)	Identity (%)	Pfam Domain(s)	VACV Core Gene	Yalta virus Ortholog(s)*	Early Promoter	Putative Function
DLEV120	156608	156441	56									Yes	Virulence: Early Promoter
DLEV121	157195	156662	178	unknown protein	XP_022536439.1	*Astyanax mexicanus*	1333	32					Unknown
DLEV122	158380	157514	289	BRO protein	YP_003517810.1	Lymantria xylina nucleopolyhedrovirus	249	32	Bro-N				Virulence: BRO Genes
DLEV123	159252	158443	270	BRO protein	AXU41742.1	Spodoptera eridania nucleopolyhedrovirus	281	33	Bro-N			Yes	Virulence: BRO Genes
DLEV124	159401	159817	139										Unknown
DLEV125	159858	160055	66								Yalta_101	Yes	Virulence: Early Promoter
DLEV126	160511	160044	156	IMV entry/fusion membrane protein	YP_008658542.1	Squirrelpox virus	140	35	Pox_A28	A28L	Yalta_098		Structure/Morphogenesis
DLEV127	160835	160548	96										Unknown
DLEV128	160852	162750	633	poly(A) polymerase large subunit	NP_064820.1	Amsacta moorei entomopoxvirus	573	27	Pox_polyA_pol	E1L	Yalta_100		Transcription/RNA Modification
DLEV129	164286	164474	63								Yalta_097		Unknown
DLEV130	164509	165717	403								Yalta_068		Unknown
DLEV131	166774	167670	299									Yes	Virulence: Early Promoter
DLEV132	168566	167835	244	myristylated membrane protein	YP_009046359.1	Pigeonpox virus	243	37	L1R_F9L	L1R	Yalta_028		Structure/Morphogenesis
DLEV133	168788	168567	74										Unknown
DLEV134	168896	172432	1179	RNA polymerase 132kDa subunit (RPO132)	NP_048226.1	Melanoplus sanguinipes entomopoxvirus	1190	31	RNA_pol_Rpb2_6, RNA_pol_Rpb2_7	A24R	Yalta_051		Transcription/RNA Modification
DLEV135	172457	172753	99										Unknown
DLEV136	172830	173780	317						SWIB			Yes	Virulence: Early Promoter
DLEV137	176579	176247	111										Unknown
DLEV138	177397	176570	276	mRNA decapping enzyme	YP_008003878.1	Adoxophyes honmai entomopoxvirus	282	31		D10R	Yalta_047		Transcription/RNA Modification
DLEV139	179651	177366	762	RAP94 RNA polymerase-associated transcriptional specificity factor	YP_008004477.1	Choristoneura rosaceana entomopoxvirus	822	22	Pox_Rap94	H4L	Yalta_046		Transcription/RNA Modification
DLEV140	179729	180856	376									Yes	Virulence: Early Promoter
DLEV141	181188	182129	314	unknown protein	AUS94114.1	Trichoplusia ni ascovirus 6b	344	28	DUF4419				Unknown
DLEV142	182145	183152	336	DNA topoisomerase type I	NP_064834.1	Amsacta moorei entomopoxvirus	333	49	Topoisom_I, VirDNA-topo-I_N	H6R	Yalta_041		Transcription/RNA Modification
DLEV143	183136	183531	132										Unknown
DLEV144	186023	184968	352	unknown protein	XP_018020522.1	*Hyalella azteca*	339	41	HAUS2				Unknown

Locus Tag	Start	End	Size (aa)	Gene ID	Best BLAST Hit				Pfam Domain(s)	VACV Core Gene	Yalta virus Ortholog(s)*	Early Promoter	Putative Function
					Accession	Source Species	Size (aa)	Identity (%)					
DLEV145	186220	188655	812	mRNA capping enzyme large subunit	NP_064917.1	Amsacta moorei entomopoxvirus	872	28	Pox_MCEL, Pox_ATPase-GT	D1R	Yalta_038		Transcription/RNA Modification
DLEV146	188722	190053	444	unknown protein	XP_005059778.1	*Ficedula albicollis*	593	44	DUF4573		Yalta_037	Yes	Virulence: Early Promoter
DLEV147	192522	190579	648	P4b precursor	YP_004821457.1	Yokapox virus	646	26	Pox_P4B	A3L	Yalta_036		Structure/Morphogenesis
DLEV148	192529	193305	259	viral late transcription factor (VLTF2)	NP_064829.1	Amsacta moorei entomopoxvirus	259	36	PLATZ	A1L	Yalta_035		Transcription/RNA Modification
DLEV149	195149	194508	214	viral late transcription factor (VLTF3)	YP_008003748.1	Mythimna separata entomopoxvirus	228	30	Pox_VLTF3, A2L_zn_ribbon	A2L	Yalta_031		Transcription/RNA Modification
DLEV150	195170	196810	547	rifampicin resistance protein	YP_009001666.1	Anomala cuprea entomopoxvirus	566	30	Pox_Rif	D13L	Yalta_030		Structure/Morphogenesis
DLEV151	196811	197368	186										Unknown
DLEV152	198686	198309	126										Unknown
DLEV153	199108	198689	140						Hormone_recep				Unknown
DLEV154	199110	199592	161										Unknown
DLEV155	200069	199557	171										Unknown
DLEV156	200613	201146	178									Yes	Virulence: Early Promoter
DLEV157	201995	201657	113									Yes	Virulence: Early Promoter
DLEV158	202722	206045	1108	DNA topoisomerase type II	XP_004994772.1	*Salpingoeca rosetta*	1795	38	DNA_topoisoIV, TOPRIM_C, DNA_gyraseB, HATPase_c, Toprim		Yalta_014	Yes	Transcription/RNA Modification
DLEV159	206800	207879	360								Yalta_111		Unknown
DLEV160	207906	209990	695	RNA helicase/nucleoside triphosphatase II	YP_008003621.1	Mythimna separata entomopoxvirus	714	33	Helicase_C, NPH-II, DEAD	I8R	Yalta_112		Transcription/RNA Modification
DLEV161	211289	211537	83	unknown protein	WP_077952852.1	*Listeria monocytogenes*	333	60					Unknown
DLEV162	212457	211567	297								Yalta_022		Unknown
DLEV163	212566	213138	191						IF4E		Yalta_114		Unknown
DLEV164	213165	214532	456	unknown protein	WP_056620920.1	*Brevundimonas sp.* Root1423	398	25	LCAT		Yalta_113		Unknown
DLEV165	215387	215058	110	FAD-linked sulfhydryl oxidase	NP_048164.1	Melanoplus sanguinipes entomopoxvirus	107	40	Pox_E10	E10R	Yalta_110		Structure/Morphogenesis
DLEV166	215413	215808	132	ATP-dependent DNA ligase	NP_064902.1	Amsacta moorei entomopoxvirus	140	24			Yalta_109		Unknown
DLEV167	217252	216371	294	poly(A) polymerase small subunit	YP_008004175.1	Choristoneura biennis entomopoxvirus	294	45	PARP_regulatory	J3R	Yalta_108	Yes	Transcription/RNA Modification

					Best BLAST Hit								
Locus Tag	Start	End	Size (aa)	Gene ID	Accession	Source Species	Size (aa)	Identity (%)	Pfam Domain(s)	VACV Core Gene	Yalta virus Ortholog(s)*	Early Promoter	Putative Function
DLEV168	217277	221026	1250	DNA polymerase	YP_005296243.1	Cotia virus SPAn232	1006	30	DNA_pol_B, DNA_pol_B_3	E9L	Yalta_107	Yes	Replication
DLEV169	222817	224868	684	nucleoside triphosphatase/helicase 2	YP_008004504.1	Choristoneura rosaceana entomopoxvirus	867	42	IBR			Yes	Transcription/RNA Modification
DLEV170	225374	224961	138										Unknown
DLEV171	226388	225381	336	unknown protein	WP_080051845.1	*Oceanospirillum multiglobuliferum*	970	44	RhoGEF				Unknown
DLEV172	226454	226675	74						T_hemolysin				Virulence: Homology
DLEV173	226683	227102	140									Yes	Virulence: Early Promoter
DLEV174	227136	228014	293									Yes	Virulence: Early Promoter
DLEV175	228507	229199	231	BRO protein	WP_002988317.1	*Streptococcus pyogenes*	253	31	Bro-N				Virulence: BRO Genes
DLEV176	229254	229910	219	thymidylate kinase	XP_016910867.1	*Apis cerana*	214	44	Thymidylate_kin			Yes	Virulence: Homology
DLEV177	229964	230605	214	BRO protein	YP_008004340.1	Choristoneura biennis entomopoxvirus	220	33	Bro-N				Virulence: BRO Genes
DLEV178	231583	230978	202	deoxynucleoside kinase	XP_024870688.1	*Temnothorax curvispinosus*	241	50	dNK		Yalta_042, Yalta_119	Yes	Virulence: Homology
DLEV179	231652	232365	238	F-box protein	XP_011633068.1	*Pogonomyrmex barbatus*	354	28	FBA, F-box-like				Virulence: Homology
DLEV180	232428	232826	133	BRO protein	AYN44984.1	Spodoptera exigua multiple nucleopolyhedrovirus	473	31	Bro-N			Yes	Virulence: BRO Genes
DLEV181	233229	233675	149	BRO protein	YP_009221130.1	Diadromus pulchellus ascovirus 4a	343	30	Bro-N				Virulence: BRO Genes
DLEV182	233770	234213	148	BRO protein	YP_009001476.1	Anomala cuprea entomopoxvirus	394	32	Bro-N				Virulence: BRO Genes
DLEV183	234309	234809	167									Yes	Virulence: Early Promoter
DLEV184	235205	235672	156										Unknown

*Orthologs between DlEPV and Yalta virus were identified with OrthoFinder v2.3.7 [1] and additional blastp searches between the two genomes.

Reference

1. Emms DM, Kelly S. OrthoFinder: solving fundamental biases in whole genome comparisons dramatically improves orthogroup inference accuracy. Genome Biol. 2015;16: 157.
