# Supplementary material for: Genomic analysis reveals an exogenous viral symbiont with dual functionality in parasitoid wasps and their hosts
Source: PLoS Pathog. 2020 Nov 30;16(11):e1009069. doi: 10.1371/journal.ppat.1009069 (PMC7728225; doi:10.1371/journal.ppat.1009069)
Supplement: S2 Table — (PDF) [file ppat.1009069.s002.pdf]

S2 Table. Sequenced EPV genome features.

Genome	ORFs (#)	Total Length (bp)	Average ITR Length (bp)	GC (%)	Coding Density (%)
AMEV	294	232,392	9,458	17.8	95.5
AHEV	247	228,750	5,617	21.0	92.0
CBEV	334	307,691	23,817	19.7	87.0
CREV	296	282,895	13,406	19.5	87.8
MySEV	306	281,182	7,347	19.7	88.6
ACEV	263	245,717	22, 978	20.0	89.8
MSEV	267	236,120	7,201	18.3	91.7
LHEV (partial)	53	46,321	N/A	23.6	90.4
Yalta virus	177	219,929	8,403	25.2	85.8
DlEPV	193	252,940	17,469	30.1	65.1
