# Supplementary material for: Genomic analysis reveals an exogenous viral symbiont with dual functionality in parasitoid wasps and their hosts
Source: PLoS Pathog. 2020 Nov 30;16(11):e1009069. doi: 10.1371/journal.ppat.1009069 (PMC7728225; doi:10.1371/journal.ppat.1009069)
Supplement: S3 Table — The 49 poxvirus core genes are shown with corresponding locus tags for homologous ORFs in EPV and CPV genomes. The 16 core genes used to build the phylogeny in Fig 2 are highlighted in yellow. (PDF) [file ppat.1009069.s003.pdf]

S3 Table. Poxvirus core gene homologs in EPV and CPV genomes. The 49 poxvirus core genes are shown with corresponding locus tags for homologous ORFs in EPV and CPV genomes. The 16 core genes used to build the phylogeny in Fig 2 are highlighted in yellow.

Gene Function	Gene Name	MSEV	ACEV	AMEV	AHEV	CREV	MySEV	Yalta virus	LHEV	DlEPV	VACV	ORFV	CRV	FPV	MOCV	SGPV
S-S bond formation pathway protein substrate	F9L	MseVgp094	ACV218	AMV243	AHEV_221	CHREV_246	MYSEV_268	Yalta_151		DLEV040	VACWR048	ORFVgORF131	CRV036	FPV112	MOCVgp016	AL387_gp092
Essential Ser/Thr kinase morph	F10L	MseVgp173	ACV137	AMV153	AHEV_178	CHREV_166	MYSEV_192	Yalta_093	EXJ30_gp10	DLEV021	VACWR049	ORFVgORF130	CRV037	FPV111	MOCVgp017	AL387_gp067
Poly(A) polymerase catalytic subunit VP55	E1L	MseVgp143	ACV146	AMV038	AHEV_038	CHREV_064	MYSEV_064	Yalta_100	EXJ30_gp07	DLEV128	VACWR057	ORFVgORF018	CRV054	FPV102	MOCVgp031	AL387_gp070
Virion protein	E6R	MseVgp145	ACV163	AMV170	AHEV_163	CHREV_181	MYSEV_213				VACWR062	ORFVgORF022	CRV056	FPV096	MOCVgp037	AL387_gp074
DNA polymerase	E9L	MseVgp036	ACV226	AMV050	AHEV_049	CHREV_048	MYSEV_075	Yalta_107		DLEV168	VACWR065	ORFVgORF025	CRV059	FPV094	MOCVgp039	AL387_gp076
Sulfhydryl oxidase, FAD linked	E10R	MseVgp093	ACV068	AMV114	AHEV_103	CHREV_129	MYSEV_170	Yalta_110	LHEV_012	DLEV165	VACWR066	ORFVgORF026	CRV060	FPV093	MOCVgp040	AL387_gp077
Virion core cysteine protease	I7L	MseVgp189	ACV108	AMV181	AHEV_156	CHREV_191	MYSEV_217	Yalta_147		DLEV042	VACWR076	ORFVgORF035	CRV070	FPV083	MOCVgp049	AL387_gp151
RNA helicase, DExH-NPH-II domain	I8R	MseVgp086	ACV170	AMV081	AHEV_079	CHREV_106	MYSEV_104	Yalta_112	LHEV_013	DLEV160	VACWR077	ORFVgORF036	CRV071	FPV082	MOCVgp050	AL387_gp150
Metalloprotease	G1L	MseVgp056	ACV184	AMV256	AHEV_231	CHREV_258	MYSEV_027	Yalta_131	LHEV_016	DLEV026	VACWR078	ORFVgORF037	CRV072	FPV081	MOCVgp056	AL387_gp149
FEN1-like nuclease	G5R	MseVgp115	ACV109	AMV179	AHEV_157	CHREV_186	MYSEV_215	Yalta_145		DLEV044	VACWR082	ORFVgORF041	CRV076	FPV117	MOCVgp060	AL387_gp146
NlpC/P60 superfamily protein	G6R	MseVgp039	ACV228	AMV041	AHEV_040	CHREV_061	MYSEV_066	Yalta_021			VACWR084	ORFVgORF043	CRV078	FPV119	MOCVgp062	AL387_gp144
Entry-fusion complex component, myristylprotein	G9R	MseVgp121	ACV056	AMV035	AHEV_036	CHREV_067	MYSEV_060	Yalta_067		DLEV062	VACWR087	ORFVgORF046	CRV081	FPV127	MOCVgp068	AL387_gp141
IMV membrane protein	L1R	MseVgp183	ACV097	AMV217	AHEV_135	CHREV_211	MYSEV_240	Yalta_028	EXJ30_gp03	DLEV132	VACWR088	ORFVgORF047	CRV082	FPV128	MOCVgp069	AL387_gp097
Internal virion protein	L3L	MseVgp180	ACV144	AMV069	AHEV_066	CHREV_095	MYSEV_092	Yalta_060		DLEV061	VACWR090	ORFVgORF049	CRV092	FPV130	MOCVgp072	AL387_gp066
ssDNA/dsDNA binding protein VP8	L4R	MseVgp158	ACV096	AMV061	AHEV_061	CHREV_083	MYSEV_084	Yalta_059		DLEV036	VACWR091	ORFVgORF050	CRV093	FPV131	MOCVgp073	AL387_gp065
Entry and fusion IMV protein	L5R	MseVgp129	ACV037	AMV083	AHEV_081	CHREV_108	MYSEV_106	Yalta_096		DLEV060	VACWR092	ORFVgORF051	CRV094	FPV132	MOCVgp074	AL387_gp064
Poly(A) polymerase small subunit VP39	J3R	MseVgp041	ACV180	AMV060	AHEV_057	CHREV_082	MYSEV_083	Yalta_108		DLEV167	VACWR095	ORFVgORF053	CRV096	FPV134	MOCVgp076	AL387_gp062
Putative late 16-kDa membrane protein	J5L	MseVgp142	ACV042	AMV232	AHEV_211	CHREV_235	MYSEV_258	Yalta_056		DLEV035	VACWR097	ORFVgORF055	CRV098	FPV136	MOCVgp078	AL387_gp059
RNA polymerase RPO147	J6R	MseVgp043	ACV230	AMV221	AHEV_199	CHREV_222	MYSEV_244	Yalta_105		DLEV067	VACWR098	ORFVgORF056	CRV099	FPV137	MOCVgp079	AL387_gp165
Entry-fusion complex essential component	H2R	MseVgp060	ACV178	AMV127	AHEV_194	CHREV_149	MYSEV_156	Yalta_125		DLEV066	VACWR100	ORFVgORF058	CRV101	FPV139	MOCVgp083	AL387_gp168

IMV heparin binding surface protein	H3L	MseVgp206	ACV112	AMV248	AHEV_225	CHREV_252	MYSEV_274	Yalta_103, Yalta_115*			VACWR101	ORFVgORF059	CRV103	FPV140	MOCVgp084	
RNA polymerase-associated protein RAP94	H4L	MseVgp118	ACV150	AMV054	AHEV_054	CHREV_073	MYSEV_079	Yalta_046	EXJ30_gp02	DLEV139	VACWR102	ORFVgORF060	CRV104	FPV141	MOCVgp085	AL387_gp169
DNA topoisomerase type I	H6R	MseVgp130	ACV152	AMV052	AHEV_052	CHREV_071	MYSEV_077	Yalta_041	LHEV_004, LHEV_005**	DLEV142	VACWR104	ORFVgORF062	CRV106	FPV143	MOCVgp087	AL387_gp171
mRNA-capping enzyme large subunit	D1R	MseVgp067	ACV140	AMV135	AHEV_190	CHREV_154	MYSEV_178	Yalta_038	EXJ30_gp04	DLEV145	VACWR106	ORFVgORF064	CRV109	FPV146	MOCVgp090	AL387_gp173
Uracil-DNA glycosylase, DNA polymerase processivity factor	D4R	MseVgp208	ACV039	AMV231	AHEV_210	CHREV_234	MYSEV_257			DLEV047	VACWR109	ORFVgORF067	CRV112	FPV062	MOCVgp093	AL387_gp134
NTPase, DNA primase	D5R	MseVgp089	ACV164	AMV087	AHEV_085	CHREV_112	MYSEV_110	Yalta_152		DLEV039	VACWR110	ORFVgORF068	CRV113	FPV058	MOCVgp094	AL387_gp091
Morph, early transcription factor small subunit (VETF-s)	D6R	MseVgp113	ACV110	AMV174	AHEV_159	CHREV_185	MYSEV_214	Yalta_024		DLEV024	VACWR111	ORFVgORF069	CRV114	FPV057	MOCVgp095	AL387_gp090
RNA polymerase RPO18	D7R	MseVgp245	ACV044	AMV230	AHEV_207	CHREV_232	MYSEV_253	Yalta_120		DLEV029	VACWR112	ORFVgORF070	CRV115	FPV056	MOCVgp097	AL387_gp136
mRNA-decapping enzyme	D10R	MseVgp150	ACV147	AMV058	AHEV_055	CHREV_080	MYSEV_081	Yalta_047		DLEV138	VACWR115	ORFVgORF071	CRV117	FPV053	MOCVgp099	AL387_gp137
ATPase, NPH1	D11R	MseVgp053	ACV074	AMV192	AHEV_150	CHREV_196	MYSEV_222	Yalta_079	EXJ30_gp08, LHEV_041**	DLEV081	VACWR116	ORFVgORF072	CRV118	FPV052	MOCVgp100	AL387_gp138
mRNA-capping enzyme small subunit	D12R	MseVgp124	ACV049	AMV093	AHEV_090	CHREV_117	MYSEV_115	Yalta_143		DLEV034	VACWR117	ORFVgORF074	CRV119	FPV051	MOCVgp101	AL387_gp140
Trimeric virion coat protein; rifampin resistance	D13L	MseVgp069	ACV193	AMV122	AHEV_112	CHREV_139	MYSEV_176	Yalta_030	EXJ30_gp05	DLEV150	VACWR118	ORFVgORF075	CRV120	FPV050	MOCVgp102	AL387_gp098
Late transcription factor (VLTF-2)	A1L	MseVgp187	ACV093	AMV047	AHEV_046	CHREV_056	MYSEV_072	Yalta_035	LHEV_026	DLEV148	VACWR119	ORFVgORF076	CRV121	FPV049	MOCVgp103	AL387_gp099
Late transcription factor (VLTF-3)	A2L	MseVgp065	ACV192	AMV205	AHEV_142	CHREV_203	MYSEV_231	Yalta_031	LHEV_027	DLEV149	VACWR120	ORFVgORF077	CRV122	FPV165	MOCVgp104	AL387_gp100
P4b precursor	A3L	MseVgp164	ACV131	AMV147	AHEV_182	CHREV_162	MYSEV_186	Yalta_036	LHEV_035	DLEV147	VACWR122	ORFVgORF079	CRV124	FPV167	MOCVgp106	AL387_gp101
RNA polymerase RPO19	A5R	MseVgp101	ACV100	AMV166	AHEV_168	CHREV_176	MYSEV_202	Yalta_133		DLEV055	VACWR124	ORFVgORF081	CRV126	FPV169	MOCVgp108	AL387_gp103
Early transcription factor large subunit (VETF-L)	A7L	MseVgp063	ACV187	AMV105	AHEV_106	CHREV_127	MYSEV_121	Yalta_140		DLEV064	VACWR126	ORFVgORF083	CRV128	FPV171	MOCVgp110	AL387_gp105
Viral membrane-associated early morphogenesis protein	A9L	MseVgp108	ACV104	AMV161	AHEV_173	CHREV_171	MYSEV_197	Yalta_141		DLEV063	VACWR128	ORFVgORF085	CRV130	FPV173	MOCVgp112	AL387_gp107
P4a precursor	A10R	MseVgp152	ACV143	AMV139	AHEV_187	CHREV_157	MYSEV_181	Yalta_071	EXJ30_gp09	DLEV111	VACWR129	ORFVgORF086	CRV131	FPV174	MOCVgp113	AL387_gp108
Viral membrane formation	A11R	MseVgp151	ACV142	AMV138	AHEV_188	CHREV_156	MYSEV_180	Yalta_072		DLEV110	VACWR130	ORFVgORF087	CRV132	FPV175	MOCVgp114	AL387_gp110
Myristylated protein, essential for entry/fusion	A16L	MseVgp090	ACV066	AMV118	AHEV_099	CHREV_133	MYSEV_131	Yalta_134		DLEV046	VACWR136	ORFVgORF093	CRV139	FPV181	MOCVgp121	AL387_gp118
DNA helicase, transcript release factor	A18R	MseVgp148	ACV148	AMV059	AHEV_056	CHREV_081	MYSEV_082	Yalta_075		DLEV078	VACWR138	ORFVgORF095	CRV141	FPV183	MOCVgp123	AL387_gp120

IMV membrane protein entry/fusion complex component	A21L	MseVgp209	ACV040	AMV249	AHEV_226	CHREV_253	MYSEV_275	Yalta_138		DLEV049	VACWR140	ORFVgORF098	CRV144	FPV186	MOCVgp125	AL387_gp122
Holliday junction resolvase	A22R	MseVgp106	ACV103	AMV162	AHEV_172	CHREV_172	MYSEV_198	Yalta_069		DLEV113	VACWR142	ORFVgORF099	CRV145	FPV187	MOCVgp127	AL387_gp124
Intermediate transcription factor 45-kDa subunit (VITF-3)	A23R	MseVgp052	ACV190	AMV091	AHEV_089	CHREV_116	MYSEV_114	Yalta_095		DLEV059	VACWR143	ORFVgORF100	CRV146	FPV188	MOCVgp128	AL387_gp125
RNA polymerase RPO132	A24R	MseVgp155	ACV085	AMV066	AHEV_068	CHREV_097	MYSEV_094	Yalta_051		DLEV134	VACWR144	ORFVgORF101	CRV147	FPV189	MOCVgp129	AL387_gp126
IMV MP/virus entry	A28L	MseVgp132	ACV071	AMV186	AHEV_152	CHREV_194	MYSEV_220	Yalta_098		DLEV126	VACWR151	ORFVgORF105	CRV149	FPV192	MOCVgp134	AL387_gp127
RNA polymerase RPO35	A29L	MseVgp149	ACV154	AMV051	AHEV_051	CHREV_070	MYSEV_076				VACWR152	ORFVgORF106	CRV150	FPV193	MOCVgp135	AL387_gp128
ATPase/DNA-packaging protein	A32L	MseVgp171	ACV135	AMV150	AHEV_180	CHREV_164	MYSEV_190	Yalta_094	EXJ30_gp11	DLEV045	VACWR155	ORFVgORF108	CRV154	FPV197	MOCVgp140	AL387_gp068

*Homolog has two full-length copies within the genome.

**Homolog is split between two adjacent putative ORFs, likely a frameshift sequencing error.
