# Supplementary material for: Genomic analysis reveals an exogenous viral symbiont with dual functionality in parasitoid wasps and their hosts
Source: PLoS Pathog. 2020 Nov 30;16(11):e1009069. doi: 10.1371/journal.ppat.1009069 (PMC7728225; doi:10.1371/journal.ppat.1009069)
Supplement: S4 Table — BRO genes are defined as those with a Bro-N protein domain. Protein domains were identified using hmmsearch to query genes from each genome against the Pfam database. A maximum e-value cutoff of 0.05 was used to isolate significant domain matches. (PDF) [file ppat.1009069.s004.pdf]

S4 Table. BRO genes in EPV genomes. BRO genes are defined as those with a Bro-N protein domain. Protein domains were identified using hmmsearch to query genes from each genome against the Pfam database. A maximum *e*-value cutoff of 0.05 was used to isolate significant domain matches.

DlEPV	ACEV	CBEV	CREV	Yalta virus	MSEV	AMEV	MySEV	AHEV
DLEV003	ACV003	CHBEV_089	CHREV_068	Yalta_004	MseVgp023	AMV055	MYSEV_087	N/A
DLEV012	ACV004	CHBEV_091	CHREV_069	Yalta_012	MseVgp024	AMV057		
DLEV014	ACV005	CHBEV_092	CHREV_075	Yalta_025	MseVgp025	AMV175		
DLEV082	ACV006	CHBEV_093	CHREV_076	Yalta_026	MseVgp194	AMV177		
DLEV083	ACV011	CHBEV_099	CHREV_077	Yalta_091	MseVgp195	AMV259		
DLEV084	ACV032	CHBEV_100	CHREV_078	Yalta_123	MseVgp196	AMV262		
DLEV092	ACV198	CHBEV_101	CHREV_079	Yalta_126	MseVgp204			
DLEV094	ACV253	CHBEV_102	CHREV_107	Yalta_127	MseVgp226			
DLEV096	ACV258	CHBEV_139	CHREV_190	Yalta_129	MseVgp229			
DLEV097	ACV259	CHBEV_222	CHREV_237	Yalta_174				
DLEV098	ACV260	CHBEV_270	CHREV_263					
DLEV100	ACV261	CHBEV_297						
DLEV101								
DLEV103								
DLEV105								
DLEV107								
DLEV109								
DLEV118								
DLEV119								
DLEV122								
DLEV123								
DLEV175								
DLEV177								
DLEV180								
DLEV181								
DLEV182								
DLEV191								
