# Supplementary material for: Genomic analysis reveals an exogenous viral symbiont with dual functionality in parasitoid wasps and their hosts
Source: PLoS Pathog. 2020 Nov 30;16(11):e1009069. doi: 10.1371/journal.ppat.1009069 (PMC7728225; doi:10.1371/journal.ppat.1009069)
Supplement: S5 Table — Genes are grouped by their putative function based on similarity to other poxvirus genes. Genes of putative virulence function are subdivided between those with a Bro-N domain (Virulence: BRO Genes), those that were identified by sequence similarity to known virulence genes (Virulence: Homology), and those that have a conserved EPV early gene promoter motif and no other assigned function (Virulence: Early Promoter). Genes with an asterisk indicate those that demonstrated significant differential expression between the two treatments (q < 0.05). (PDF) [file ppat.1009069.s005.pdf]

S5 Table. Expression of DlEPV genes in wasp venom gland (DL) and parasitized fly (AS) samples. Genes are grouped by their putative function based on similarity to other poxvirus genes. Genes of putative virulence function are subdivided between those with a Bro-N domain (Virulence: BRO Genes), those that were identified by sequence similarity to known virulence genes (Virulence: Homology), and those that have a conserved EPV early gene promoter motif and no other assigned function (Virulence: Early Promoter). Genes with an asterisk indicate those that demonstrated significant differential expression between the two treatments ($q < 0.05$).

Putative Function	Gene ID	AS Mean FPKM ± SD	DL Mean FPKM ± SD	Log2 Fold Change DL over AS
DNA Replication	DLEV007*	1438 ± 334	178 ± 81	-3.01
	DLEV028*	1888 ± 832	388 ± 110	-2.28
	DLEV031*	7825 ± 5003	236 ± 64	-5.05
	DLEV039	2133 ± 858	1941 ± 176	-0.14
	DLEV047*	624 ± 75	65 ± 16	-3.27
	DLEV072*	1925 ± 1243	70 ± 10	-4.79
	DLEV113*	1742 ± 496	2775 ± 334	0.67
	DLEV168*	543 ± 292	64 ± 13	-3.08
	DLEV187*	1496 ± 365	182 ± 70	-3.04
Structure/Morphogenesis	DLEV021*	350 ± 138	1870 ± 182	2.42
	DLEV026*	1021 ± 468	6692 ± 720	2.71
	DLEV035*	768 ± 248	3867 ± 283	2.33
	DLEV036*	14619 ± 7020	70321 ± 6932	2.27
	DLEV040*	1742 ± 262	4966 ± 404	1.51
	DLEV042*	1315 ± 609	14803 ± 1011	3.49
	DLEV044*	4004 ± 3039	68 ± 19	-5.89
	DLEV045*	2645 ± 1210	11620 ± 989	2.14
	DLEV046*	993 ± 420	8520 ± 1038	3.10
	DLEV049*	2495 ± 1251	7288 ± 716	1.55
	DLEV060*	1057 ± 554	5776 ± 617	2.45

	DLEV062*	1035 ± 399	7466 ± 778	2.85
	DLEV063*	392 ± 169	957 ± 142	1.29
	DLEV066*	2105 ± 971	8023 ± 941	1.93
	DLEV110	472 ± 131	462 ± 72	-0.03
	DLEV111*	12026 ± 5775	55952 ± 12475	2.22
	DLEV126*	572 ± 186	5711 ± 473	3.32
	DLEV132*	3562 ± 1528	33045 ± 4142	3.21
	DLEV147*	12645 ± 5449	91623 ± 13910	2.86
	DLEV150*	10858 ± 4329	34698 ± 5021	1.68
	DLEV165*	222 ± 103	837 ± 123	1.91
Transcription/RNA Modification	DLEV019	836 ± 162	1036 ± 126	0.31
	DLEV020*	1756 ± 780	182 ± 22	-3.27
	DLEV024*	1120 ± 557	13183 ± 1672	3.56
	DLEV029*	3826 ± 851	8610 ± 638	1.17
	DLEV034*	767 ± 258	4126 ± 473	2.43
	DLEV055*	1752 ± 189	4903 ± 191	1.48
	DLEV058*	2977 ± 2156	457 ± 65	-2.70
	DLEV059*	1124 ± 589	434 ± 45	-1.37
	DLEV061*	1617 ± 710	21486 ± 1040	3.73
	DLEV064*	540 ± 265	3762 ± 407	2.80
	DLEV067*	1392 ± 185	3709 ± 736	1.41
	DLEV076*	2656 ± 708	223 ± 50	-3.57
	DLEV078*	780 ± 128	1291 ± 103	0.73
	DLEV081*	1312 ± 559	7886 ± 1689	2.59
	DLEV128*	1467 ± 583	5137 ± 559	1.81
	DLEV134	1884 ± 257	2225 ± 489	0.24
	DLEV138*	1337 ± 160	4137 ± 487	1.63

	DLEV139*	482 ± 207	4077 ± 695	3.08
	DLEV142*	405 ± 203	1335 ± 213	1.72
	DLEV145*	1119 ± 118	3734 ± 692	1.74
	DLEV148*	2855 ± 1830	792 ± 64	-1.85
	DLEV149*	879 ± 268	2114 ± 389	1.27
	DLEV158*	1021 ± 283	379 ± 64	-1.43
	DLEV160*	792 ± 356	8267 ± 1069	3.38
	DLEV167*	1652 ± 485	4073 ± 267	1.30
	DLEV169*	311 ± 163	27 ± 4	-3.52
Virulence: BRO Genes	DLEV003*	895 ± 495	222 ± 60	-2.01
	DLEV012*	2401 ± 594	478 ± 100	-2.33
	DLEV014*	2983 ± 1187	1937 ± 169	-0.62
	DLEV082*	1920 ± 1193	646 ± 70	-1.57
	DLEV083*	4930 ± 3859	84 ± 27	-5.87
	DLEV084	42 ± 15	37 ± 19	-0.16
	DLEV092*	3003 ± 2175	39 ± 11	-6.27
	DLEV094*	2288 ± 1359	113 ± 24	-4.34
	DLEV096*	283 ± 139	59 ± 9	-2.27
	DLEV097*	7224 ± 7206	56 ± 18	-7.00
	DLEV098*	2860 ± 2646	62 ± 12	-5.52
	DLEV100*	3295 ± 2806	42 ± 12	-6.30
	DLEV101*	4197 ± 2654	239 ± 38	-4.13
	DLEV103*	5095 ± 3453	768 ± 216	-2.73
	DLEV105*	993 ± 655	88 ± 13	-3.50
	DLEV107*	13867 ± 8198	1411 ± 286	-3.30
	DLEV109*	1154 ± 933	126 ± 23	-3.19
	DLEV118*	348 ± 36	43 ± 8	-3.02

	DLEV119*	6647 ± 4480	61 ± 17	-6.77
	DLEV122	806 ± 254	958 ± 112	0.25
	DLEV123*	1089 ± 207	260 ± 27	-2.07
	DLEV175	630 ± 405	572 ± 98	-0.14
	DLEV177*	1205 ± 224	2065 ± 151	0.78
	DLEV180*	8179 ± 7186	1177 ± 135	-2.80
	DLEV181*	2350 ± 386	7247 ± 1179	1.62
	DLEV182*	831 ± 106	91 ± 19	-3.19
	DLEV191*	939 ± 534	233 ± 60	-2.01
Virulence: Homology	DLEV037*	1398 ± 629	5410 ± 1325	1.95
	DLEV099*	1217 ± 828	53 ± 7	-4.53
	DLEV172*	4618 ± 2081	108 ± 26	-5.41
	DLEV176*	1265 ± 269	105 ± 10	-3.59
	DLEV178*	3509 ± 2522	81 ± 18	-5.44
	DLEV179*	1086 ± 423	166 ± 28	-2.71
Virulence: Early Promoter	DLEV006*	1613 ± 224	246 ± 40	-2.71
	DLEV008*	1013 ± 512	70 ± 8	-3.85
	DLEV009*	1959 ± 975	132 ± 57	-3.90
	DLEV011*	2457 ± 1228	285 ± 115	-3.11
	DLEV015*	1724 ± 1364	307 ± 37	-2.49
	DLEV016*	897 ± 453	164 ± 11	-2.46
	DLEV025*	2048 ± 1513	28 ± 8	-6.19
	DLEV050*	1260 ± 529	186 ± 26	-2.76
	DLEV052*	20090 ± 13456	433 ± 158	-5.54
	DLEV053*	1343 ± 624	9309 ± 706	2.79
	DLEV056*	3229 ± 1840	256 ± 47	-3.66
	DLEV065*	1307 ± 980	39 ± 8	-5.06

	DLEV073*	31730 ± 16272	179234 ± 15436	2.50
	DLEV074*	25226 ± 11820	135181 ± 9485	2.42
	DLEV086*	2726 ± 1248	203 ± 52	-3.75
	DLEV091*	2285 ± 1419	43 ± 12	-5.73
	DLEV093*	656 ± 347	58 ± 14	-3.49
	DLEV095*	6173 ± 4451	231 ± 43	-4.74
	DLEV106*	782 ± 147	138 ± 37	-2.50
	DLEV108	3517 ± 373	4170 ± 320	0.25
	DLEV120*	346 ± 271	5 ± 6	-5.99
	DLEV125	4089 ± 2024	6545 ± 488	0.68
	DLEV131*	1135 ± 482	367 ± 50	-1.63
	DLEV136*	7377 ± 3046	1612 ± 208	-2.19
	DLEV140*	25260 ± 3084	12907 ± 2659	-0.97
	DLEV146	4625 ± 434	5239 ± 734	0.18
	DLEV156*	769 ± 163	81 ± 33	-3.25
	DLEV157*	276 ± 106	57 ± 8	-2.27
	DLEV173*	5312 ± 4330	74 ± 20	-6.17
	DLEV174*	380 ± 87	586 ± 60	0.62
	DLEV183*	4210 ± 1754	290 ± 124	-3.86
	DLEV185*	2546 ± 1299	287 ± 51	-3.15
	DLEV186*	959 ± 457	73 ± 15	-3.71
	DLEV188	46 ± 14	58 ± 30	0.33
Unknown	DLEV001*	196 ± 54	813 ± 66	2.05
	DLEV002*	1935 ± 521	4464 ± 835	1.21
	DLEV004*	2171 ± 998	379 ± 74	-2.52
	DLEV005*	919 ± 408	46 ± 12	-4.32
	DLEV010*	1132 ± 226	287 ± 53	-1.98

DLEV013*	3723 ± 1820	43342 ± 7052	3.54
DLEV017*	3164 ± 1265	16411 ± 1403	2.37
DLEV018*	16627 ± 8595	101597 ± 7179	2.61
DLEV022*	1933 ± 621	5624 ± 1002	1.54
DLEV023*	2620 ± 479	6266 ± 423	1.26
DLEV027*	6878 ± 3113	98526 ± 10455	3.84
DLEV030*	1324 ± 698	5835 ± 512	2.14
DLEV032*	5909 ± 2536	229 ± 83	-4.69
DLEV033*	1866 ± 870	8067 ± 1381	2.11
DLEV038*	537 ± 84	3534 ± 532	2.72
DLEV041*	1880 ± 1197	315 ± 29	-2.58
DLEV043*	1482 ± 730	8235 ± 1030	2.47
DLEV048*	1104 ± 728	170 ± 28	-2.70
DLEV051*	1430 ± 403	3837 ± 614	1.42
DLEV054	1232 ± 903	15146 ± 2597	3.62
DLEV057*	2523 ± 1132	22138 ± 2493	3.13
DLEV068*	2048 ± 341	4670 ± 344	1.19
DLEV069*	2417 ± 1409	11355 ± 910	2.23
DLEV070*	676 ± 287	5774 ± 822	3.09
DLEV071	1351 ± 323	2852 ± 279	1.08
DLEV075*	866 ± 99	3119 ± 355	1.85
DLEV077*	31340 ± 14340	144080 ± 21496	2.20
DLEV079*	13981 ± 7310	50643 ± 5408	1.86
DLEV080*	7923 ± 4485	56621 ± 4577	2.84
DLEV085*	288 ± 114	2558 ± 203	3.15
DLEV087*	1180 ± 346	404 ± 45	-1.55
DLEV088*	1506 ± 666	814 ± 117	-0.89

DLEV089*	232 ± 53	157 ± 21	-0.56
DLEV090*	636 ± 134	104 ± 22	-2.62
DLEV102*	1065 ± 1006	28 ± 4	-5.24
DLEV104*	6478 ± 5296	418 ± 88	-3.95
DLEV112*	447 ± 295	67 ± 12	-2.74
DLEV114*	382 ± 119	8573 ± 1036	4.49
DLEV115*	1785 ± 892	3413 ± 736	0.93
DLEV116	10893 ± 4458	14406 ± 2269	0.40
DLEV117*	971 ± 233	469 ± 51	-1.05
DLEV121*	48 ± 13	19 ± 6	-1.29
DLEV124*	11722 ± 5902	82869 ± 6351	2.82
DLEV127*	19124 ± 8866	120280 ± 11603	2.65
DLEV129*	665 ± 522	2661 ± 374	2.00
DLEV130*	1423 ± 965	52692 ± 4856	5.21
DLEV133*	725 ± 388	4328 ± 729	2.58
DLEV135*	36153 ± 21678	179208 ± 15640	2.31
DLEV137	1109 ± 417	7297 ± 1620	2.72
DLEV141*	3120 ± 2466	66 ± 6	-5.55
DLEV143*	1815 ± 811	9943 ± 658	2.45
DLEV144*	1132 ± 545	4218 ± 695	1.90
DLEV151	11242 ± 3215	13742 ± 1109	0.29
DLEV152*	1116 ± 312	395 ± 48	-1.50
DLEV153*	1080 ± 440	8516 ± 652	2.98
DLEV154*	2728 ± 2027	583 ± 67	-2.23
DLEV155	265 ± 75	1799 ± 115	2.76
DLEV159*	8665 ± 3995	58607 ± 8451	2.76
DLEV161*	17283 ± 4211	2967 ± 325	-2.54

	DLEV162*	1577 ± 627	4337 ± 969	1.46
	DLEV163*	1424 ± 235	2032 ± 229	0.51
	DLEV164*	20096 ± 9137	95019 ± 10808	2.24
	DLEV166*	37168 ± 16529	234307 ± 18472	2.66
	DLEV170*	2358 ± 940	42421 ± 3997	4.17
	DLEV171*	1285 ± 530	3700 ± 904	1.53
	DLEV184*	5159 ± 3859	59 ± 25	-6.44
	DLEV189*	1204 ± 509	64 ± 14	-4.22
	DLEV190*	2236 ± 1105	346 ± 61	-2.69
	DLEV192*	1972 ± 637	4480 ± 700	1.18
	DLEV193*	204 ± 47	796 ± 84	1.96
