# Supplementary material for: Genomic analysis reveals an exogenous viral symbiont with dual functionality in parasitoid wasps and their hosts
Source: PLoS Pathog. 2020 Nov 30;16(11):e1009069. doi: 10.1371/journal.ppat.1009069 (PMC7728225; doi:10.1371/journal.ppat.1009069)
Supplement: S6 Table — Feature table of LHEV ORFs including the 11 ORFs previously annotated by Viljakainen et al. 2018 [43] (accession NC_040577). (PDF) [file ppat.1009069.s006.pdf]

S6 Table. Re-annotation of the LHEV genome segment. Feature table of LHEV ORFs including the 11 ORFs previously annotated by Viljakainen *et al.* 2018 [1] (accession NC_040577).

>Feature ref|NC_040577.1|

271	1764	gene		
			locus_tag	EXJ30_gp01
			db_xref	GeneID:41702288
271	1764	CDS		
			product	putative tryptophan repeat family protein
			protein_id	ref|YP_009552022.1|
2476	1649	gene		
			locus_tag	EXJ30_gp02
			db_xref	GeneID:41702291
2476	1649	CDS		
			product	putative RNA polymerase-associated transcription-specificity factor RAP94
			protein_id	ref|YP_009552023.1|
			note	similar to vaccinia virus H4
2564	3529	gene		
			locus_tag	LHEV_003
2564	3529	CDS		
			transl_table	1
			product	hypothetical protein
3584	4099	gene		

			gene	TOP1_1
			locus_tag	LHEV_004
3584	4099	CDS		
			transl_table	1
			gene	TOP1_1
			locus_tag	LHEV_004
			product	putative DNA topoisomerase type I
			note	similar to vaccinia virus H6
4312	4554	gene		
			gene	TOP1_2
			locus_tag	LHEV_005
4312	4554	CDS		
			transl_table	1
			gene	TOP1_2
			locus_tag	LHEV_005
			product	putative DNA topoisomerase type I
			note	similar to vaccinia virus H6
4547	4987	gene		
			locus_tag	LHEV_006
4547	4987	CDS		
			transl_table	1
			product	hypothetical protein

5726	4992	gene		
			locus_tag	EXJ30_gp03
			db_xref	GeneID:41702292
5726	4992	CDS		
			product	putative virion membrane protein M25
			protein_id	ref|YP_009552024.1|
			note	similar to vaccinia virus L1
5787	6038	gene		
			locus_tag	LHEV_008
5787	6038	CDS		
			transl_table	1
			product	hypothetical protein
7431	6040	gene		
			locus_tag	LHEV_009
7431	6040	CDS		
			transl_table	1
			locus_tag	LHEV_009
			product	putative DNA polymerase beta/AP endonuclease
7928	7398	gene		
			locus_tag	LHEV_010
7928	7398	CDS		
			transl_table	1

			locus_tag	LHEV_010
			product	putative DNA topoisomerase subunit
8363	7974	gene		
			locus_tag	LHEV_011
8363	7974	CDS		
			transl_table	1
			product	hypothetical protein
8692	8414	gene		
			locus_tag	LHEV_012
8692	8414	CDS		
			transl_table	1
			locus_tag	LHEV_012
			product	putative FAD-linked sulfhydryl oxidase
			note	similar to vaccinia virus E10
8734	10992	gene		
			gene	NPH2
			locus_tag	LHEV_013
8734	10992	CDS		
			transl_table	1
			gene	NPH2
			locus_tag	LHEV_013
			product	putative RNA helicase, NPH-II

			note	similar to vaccinia virus I8
11756	10947	gene		
			locus_tag	LHEV_014
11756	10947	CDS		
			transl_table	1
			product	hypothetical protein
12155	11811	gene		
			locus_tag	LHEV_015
12155	11811	CDS		
			transl_table	1
			product	hypothetical protein
12778	12263	gene		
			locus_tag	LHEV_016
12778	12263	CDS		
			transl_table	1
			locus_tag	LHEV_016
			product	putative metalloprotease
			note	similar to vaccinia virus G1
13522	12968	gene		
			locus_tag	LHEV_017
13522	12968	CDS		
			transl_table	1

			product	hypothetical protein
14114	13593	gene		
			locus_tag	LHEV_018
14114	13593	CDS		
			transl_table	1
			product	hypothetical protein
15457	14138	gene		
			locus_tag	EXJ30_gp04
			db_xref	GeneID:41702284
15457	14138	CDS		
			product	putative mRNA-capping enzyme catalytic subunit
			protein_id	ref|YP_009552025.1|
			note	similar to vaccinia virus D1
15484	16341	gene		
			locus_tag	LHEV_020
15484	16341	CDS		
			transl_table	1
			product	hypothetical protein
16701	16273	gene		
			locus_tag	LHEV_021
16701	16273	CDS		
			transl_table	1

			product	hypothetical protein
18431	16734	gene		
			locus_tag	EXJ30_gp05
			db_xref	GeneID:41702285
18431	16734	CDS		
			product	putative rifampicin resistance protein
			protein_id	ref|YP_009552026.1|
			note	similar to vaccinia virus D13
18494	19945	gene		
			locus_tag	LHEV_023
18494	19945	CDS		
			transl_table	1
			product	hypothetical protein
20357	19923	gene		
			locus_tag	LHEV_024
20357	19923	CDS		
			transl_table	1
			product	hypothetical protein
22882	20642	gene		
			locus_tag	LHEV_025
22882	20642	CDS		
			transl_table	1

			product	hypothetical protein
22888	23502	gene		
			gene	VLTF2
			locus_tag	LHEV_026
22888	23502	CDS		
			transl_table	1
			gene	VLTF2
			locus_tag	LHEV_026
			product	putative viral late transcription factor 2
			note	similar to vaccinia virus A1
24343	23663	gene		
			gene	VLTF3
			locus_tag	LHEV_027
24343	23663	CDS		
			transl_table	1
			gene	VLTF3
			locus_tag	LHEV_027
			product	putative viral late transcription factor 3
			note	similar to vaccinia virus A2
24384	25673	gene		
			locus_tag	LHEV_028
24384	25673	CDS		

			transl_table	1
			product	hypothetical protein
26351	25674	gene		
			locus_tag	LHEV_029
26351	25674	CDS		
			transl_table	1
			product	hypothetical protein
28110	26398	gene		
			locus_tag	EXJ30_gp06
			db_xref	GeneID:41702290
28110	26398	CDS		
			product	putative DNA ligase
			protein_id	ref|YP_009552027.1|
28763	28446	gene		
			locus_tag	LHEV_031
28763	28446	CDS		
			transl_table	1
			product	hypothetical protein
28968	28765	gene		
			locus_tag	LHEV_032
28968	28765	CDS		
			transl_table	1

			product	hypothetical protein
29374	28973	gene		
			locus_tag	LHEV_033
29374	28973	CDS		
			transl_table	1
			product	hypothetical protein
29477	29325	gene		
			locus_tag	LHEV_034
29477	29325	CDS		
			transl_table	1
			product	hypothetical protein
31570	29645	gene		
			locus_tag	LHEV_035
31570	29645	CDS		
			transl_table	1
			product	putative major core protein 4b
			note	similar to vaccinia virus A3
33305	31593	gene		
			locus_tag	EXJ30_gp07
			db_xref	GeneID:41702286
33305	31593	CDS		
			product	putative poly(A) polymerase catalytic subunit

			protein_id	ref|YP_009552028.1|
			note	similar to vaccinia virus E1
33443	33628	gene		
			locus_tag	LHEV_037
33443	33628	CDS		
			transl_table	1
			product	hypothetical protein
33600	33797	gene		
			locus_tag	LHEV_038
33600	33797	CDS		
			transl_table	1
			product	hypothetical protein
34720	34328	gene		
			locus_tag	LHEV_039
34720	34328	CDS		
			transl_table	1
			product	hypothetical protein
36085	34724	gene		
			locus_tag	EXJ30_gp08
			db_xref	GeneID:41702287
36085	34724	CDS		
			product	putative nucleoside triphosphatase I

			protein_id	ref|YP_009552029.1|
			note	similar to vaccinia virus D11
36624	36253	gene		
			locus_tag	LHEV_041
36624	36253	CDS		
			transl_table	1
			locus_tag	LHEV_041
			product	putative nucleoside triphosphatase I
			note	similar to vaccinia virus D11
36650	36970	gene		
			locus_tag	LHEV_042
36650	36970	CDS		
			transl_table	1
			product	hypothetical protein
37001	37258	gene		
			locus_tag	LHEV_043
37001	37258	CDS		
			transl_table	1
			product	hypothetical protein
37283	37660	gene		
			locus_tag	LHEV_044
37283	37660	CDS		

			transl_table	1
			product	hypothetical protein
37687	38028	gene		
			locus_tag	LHEV_045
37687	38028	CDS		
			transl_table	1
			product	hypothetical protein
38054	38401	gene		
			locus_tag	LHEV_046
38054	38401	CDS		
			transl_table	1
			product	hypothetical protein
39449	38406	gene		
			locus_tag	LHEV_047
39449	38406	CDS		
			transl_table	1
			product	hypothetical protein
39493	42060	gene		
			locus_tag	EXJ30_gp09
			db_xref	GeneID:41702294
39493	42060	CDS		
			product	putative major core protein 4a precursor

			protein_id	ref|YP_009552030.1|
			note	similar to vaccinia virus A10
42440	42057	gene		
			locus_tag	LHEV_049
42440	42057	CDS		
			transl_table	1
			product	hypothetical protein
42784	42560	gene		
			locus_tag	LHEV_050
42784	42560	CDS		
			transl_table	1
			product	hypothetical protein
43571	43410	gene		
			locus_tag	LHEV_051
43571	43410	CDS		
			transl_table	1
			product	hypothetical protein
44939	43572	gene		
			locus_tag	EXJ30_gp10
			db_xref	GeneID:41702293
44939	43572	CDS		
			product	putative serine/threonine-protein kinase 2

			protein_id	ref|YP_009552031.1|
			note	similar to vaccinia virus F10
44988	45743	gene		
			locus_tag	EXJ30_gp11
			db_xref	GeneID:41702289
44988	45743	CDS		
			product	putative protein A32
			prot_desc	similar to variola virus
			protein_id	ref|YP_009552032.1|

Reference

1. Viljakainen L, Holmberg I, Abril S, Jurvansuu J. Viruses of invasive Argentine ants from the European main supercolony: characterization, interactions and evolution. J Gen Virol. 2018;99: 1129–1140.
