# Supplementary material for: Genomic analysis reveals an exogenous viral symbiont with dual functionality in parasitoid wasps and their hosts
Source: PLoS Pathog. 2020 Nov 30;16(11):e1009069. doi: 10.1371/journal.ppat.1009069 (PMC7728225; doi:10.1371/journal.ppat.1009069)
Supplement: S1 Fig — DlEPV copy number relative to D. longicaudata copy number was estimated with qPCR for (A) adult female wasp reproductive tissues, and (B) female and male whole wasps in pupal-adult developmental stages. Venom glands and ovaries from adult females were pooled in triplicate for each biological replicate. DlEPV genome copy number was estimated using the poly(A) polymerase small subunit gene (polyAPol, DLEV167), and D. longicaudata copy number with the elongation factor alpha gene (EF1a). qPCR was performed as done previously [34]. The y-axes indicate the log10 fold change of total DlEPV genome copy number over total D. longicaudata genome copy number. Permanent integration of the DlEPV genome into the D. longicaudata genome would result in a ratio of virus to wasp copy number that is ≥ 1 for all wasp tissues, developmental stages, and sexes, which is equivalent to a log10 abundance fold change of 0. Negative log10 abundance fold change values indicate samples in which the virus to wasp copy number ratio was < 1. Each bar represents the average relative DlEPV copy number across 6 biological replicates, and error bars represent one standard error above and below the mean. (PDF) [file ppat.1009069.s007.pdf]

S1 Fig. Normalized DlEPV abundance in *D. longicaudata* wasps. DlEPV copy number relative to *D. longicaudata* copy number was estimated with qPCR for (A) adult female wasp reproductive tissues, and (B) female and male whole wasps in pupal-adult developmental stages. Venom glands and ovaries from adult females were pooled in triplicate for each biological replicate. DlEPV genome copy number was estimated using the poly(A) polymerase small subunit gene (polyAPol, DLEV167), and *D. longicaudata* copy number with the elongation factor alpha gene (EF1a). qPCR was performed as done previously [1]. The y-axes indicate the $\log_{10}$ fold change of total DlEPV genome copy number over total *D. longicaudata* genome copy number. Permanent integration of the DlEPV genome into the *D. longicaudata* genome would result in a ratio of virus to wasp copy number that is ≥ 1 for all wasp tissues, developmental stages, and sexes, which is equivalent to a $\log_{10}$ abundance fold change of 0. Negative $\log_{10}$ abundance fold change values indicate samples in which the virus to wasp copy number ratio was < 1. Each bar represents the average relative DlEPV copy number across 6 biological replicates, and error bars represent one standard error above and below the mean.

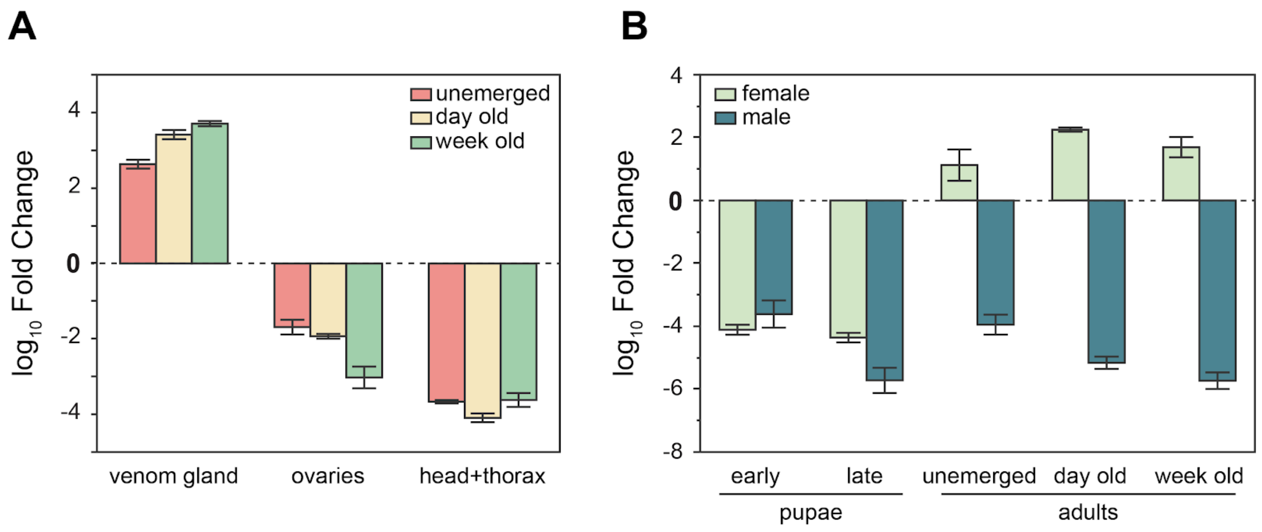

Reference

1. Coffman KA, Harrell TC, Burke GR. A mutualistic poxvirus exhibits convergent evolution with other heritable viruses in parasitoid wasps. J Virol. 2020;94: e02059–19.
