# Supplementary material for: Genomic analysis reveals an exogenous viral symbiont with dual functionality in parasitoid wasps and their hosts
Source: PLoS Pathog. 2020 Nov 30;16(11):e1009069. doi: 10.1371/journal.ppat.1009069 (PMC7728225; doi:10.1371/journal.ppat.1009069)
Supplement: S2 Fig — (A) Maximum likelihood (ML) phylogenetic tree built from a concatenated multiple sequence alignment of the 44 core genes shared by all EPV complete genomes. Methods were the same as used to build the phylogeny in Fig 2. Node support (%) was inferred with 1,000 bootstrap iterations. (B) Bayesian inference phylogeny of the concatenated 16 core genes from Fig 2 built using PhyloBayes-MPI v20161021 with the CAT-GTR substitution model [111]. Node support in panel B is labeled with the consensus posterior probability from two independent Markov chain Monte Carlo simulations that ran for 10,000 cycles each with a 1,000 cycle burn-in. (C) ML phylogeny built from a concatenated alignment of the 10 poxvirus core genes shared by all poxviruses and sister group Asfarviridae members African swine fever virus (ASFV, NC_001659) and Kaumoebavirus (NC_034249). Core genes and respective ASFV and Kaumoebavirus accession numbers used to create the phylogeny in panel C include the DNA polymerase E9L (AAA65319.1, ARA71927.1), RNA helicase I8R (AAA65302.1, ARA71975.1), RPO147 J6R (AAA65328.1, ARA71945.1 and ARA71948.1), mRNA-capping enzyme large subunit D1R (AAA65330.1, ARA71993.1), NTPase, DNA primase D5R (AAA65301.1, ARA71965.1), viral early transcription factor (VETF) small subunit D6R (AAA65335.1, ARA72203.1), VETF large subunit A7L (AAA65318.1, ARA71923.1), ATPase NPH1 D11R (AAA65350.1, ARA72259.1), RPO132 A24R (AAA65283.1, ARA72182.1), and ATPase/DNA-packaging protein A32L (AAA65308.1, ARA72015.1). Tree building methods were the same as done for other ML trees. (PDF) [file ppat.1009069.s008.pdf]

S2 Fig. Additional poxvirus core gene phylogenies. (A) Maximum likelihood (ML) phylogenetic tree built from a concatenated multiple sequence alignment of the 44 core genes shared by all EPV complete genomes. Methods were the same as used to build the phylogeny in Fig 2. Node support (%) was inferred with 1,000 bootstrap iterations. (B) Bayesian inference phylogeny of the concatenated 16 core genes from Fig 2 built using PhyloBayes-MPI v20161021 with the CAT-GTR substitution model [1]. Node support in panel B is labeled with the consensus posterior probability from two independent Markov chain Monte Carlo simulations that ran for 10,000 cycles each with a 1,000 cycle burn-in. (C) ML phylogeny built from a concatenated alignment of the 10 poxvirus core genes shared by all poxviruses and sister group Asfarviridae members African swine fever virus (ASFV, NC_001659) and Kaumoebavirus (NC_034249). Core genes and respective ASFV and Kaumoebavirus accession numbers used to build the phylogeny in panel C include the DNA polymerase E9L (AAA65319.1, ARA71927.1), RNA helicase I8R (AAA65302.1, ARA71975.1), RPO147 J6R (AAA65328.1, ARA71945.1 and ARA71948.1), mRNA-capping enzyme large subunit D1R (AAA65330.1, ARA71993.1), NTPase, DNA primase D5R (AAA65301.1, ARA71965.1), viral early transcription factor (VETF) small subunit D6R (AAA65335.1, ARA72203.1), VETF large subunit A7L (AAA65318.1, ARA71923.1), ATPase NPH1 D11R (AAA65350.1, ARA72259.1), RPO132 A24R (AAA65283.1, ARA72182.1), and ATPase/DNA-packaging protein A32L (AAA65308.1, ARA72015.1). Tree building methods were the same as done for other ML trees.

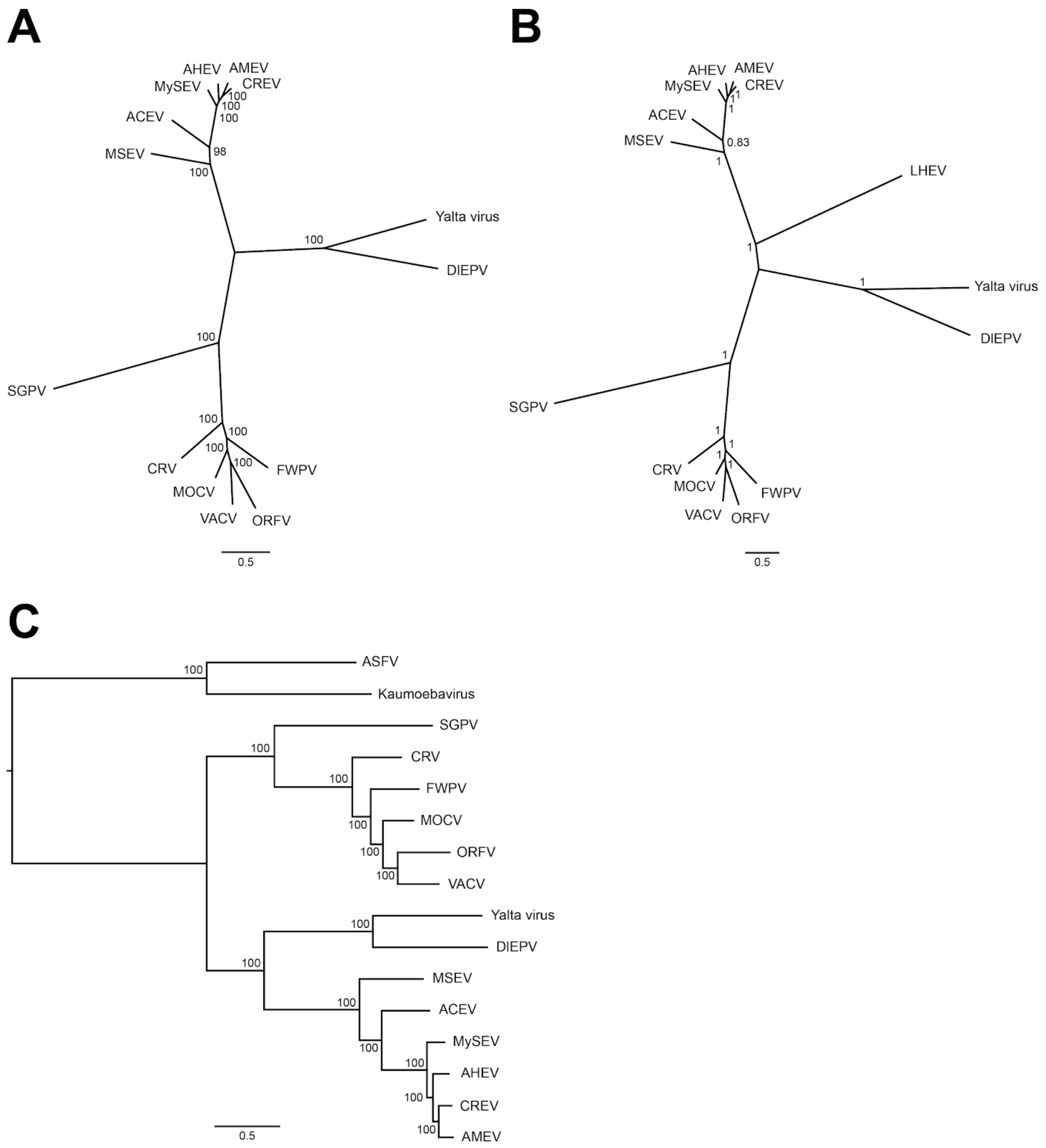

Reference

1. Lartillot N, Philippe H. A Bayesian mixture model for across-site heterogeneities in the amino-acid replacement process. Mol Biol Evol. 2004;21: 1095–1109.
